# *Cohnella* 1759 cysteine protease shows significant long term half-life and impressive increased activity in presence of some chemical reagents

**DOI:** 10.1038/s41598-021-84267-w

**Published:** 2021-02-25

**Authors:** Rayan Saghian, Elham Mokhtari, Saeed Aminzadeh

**Affiliations:** Bioprocess Engineering Group, Institute of Industrial and Environmental Biotechnology, National Institute of Genetic Engineering and Biotechnology (NIGEB), Tehran, Iran

**Keywords:** Enzymes, Proteases

## Abstract

Thermostability and substrate specificity of proteases are major factors in their industrial applications. rEla is a novel recombinant cysteine protease obtained from a thermophilic bacterium, *Cohnella* sp.A01 (PTCC No: 1921). Herein, we were interested in recombinant production and characterization of the enzyme and finding the novel features in comparison with other well-studied cysteine proteases. The bioinformatics analysis showed that rEla is allosteric cysteine protease from DJ-1/ThiJ/PfpI superfamily. The enzyme was heterologously expressed and characterized and the recombinant enzyme molecular mass was 19.38 kD which seems to be smaller than most of the cysteine proteases. rEla exhibited acceptable activity in broad pH and temperature ranges. The optimum activity was observed at 50℃ and pH 8 and the enzyme showed remarkable stability by keeping 50% of residual activity after 100 days storage at room temperature. The enzyme K_m_ and V_max_ values were 21.93 mM, 8 U/ml, respectively. To the best of our knowledge*,* in comparison with the other characterized cysteine proteases, rEla is the only reported cysteine protease with collagen specificity. The enzymes activity increases up to 1.4 times in the presence of calcium ion (2 mM) suggesting it as the enzyme’s co-factor. When exposed to surfactants including Tween20, Tween80, Triton X-100 and SDS (1% and 4% v/v) the enzyme activity surprisingly increased up to 5 times.

## Introduction

Microbial enzymes e.g. proteases are known as supreme enzymes utilized in different industries^1^. All five classes of proteases including serine, aspartate, threonine, cysteine, and metalloproteases are found and therefore exist in prokaryotes^2,3^. In comparison to other classes, the cysteine proteases seems to be introduced less in bacteria^2^, however, the presence of cysteine proteases in prokaryotes is as common as in eukaryotes^4^.

Although most of the cysteine protease members are endopeptidases, there are few members which have exopeptidase activity exclusively or additionally. Cysteine proteases are defined as proteases that in their catalysis process, the thiol group of a cysteine residue plays the role of a nucleophile^3^. These peptidases catalyze the carboxylic acid derivatives hydrolysis through a two-step pathway in which the formation of general acid–base and an acyl-thiol intermediate hydrolysis occurs^5^. Interest in cysteine peptidases is growing and they are being utilized in different industries such as medicinal applications^6^.

In order to prevent their proteolytic activity on non-substrate proteins, proteases are regulated carefully. Allostery is a reversible swift regulation of enzyme activities without energy consumption. Allosteric enzymes have a site distinct from their catalytic center which controls and regulates enzymatic activity through interaction with small molecules having activatory or inhibitory effects. Although the prominent role of allostery in regulation of different proteases has received much attention, the study of small molecules effects such as inhibitors in regulating them is still in early stages^7^.

Native collagens have a triple-helical structure that common proteases are unable to hydrolyze^8^. Collagenases are proteases which break down the peptide bonds in specific sites of collagen^9^. Bacterial collagenases due to their wide industrial and biological applications are propitious enzymes^10^. Microbial collagenases have been directly utilized in clinical treatments and in laboratory researches as experimental reactants^11^. They can tenderize meat by digesting collagens^12,13^, be used for leather dyeing^14^ and wound healing as alternatives to surgical wound debridement^15,16^ by eliminating cellular remnants and extracellular tissue necrosis^17^.

They have been employed to create animal models of acute neurological injuries^18^, repair cartilage by increasing cell density^9^ and cure Dupuytren's disease^19^. Regarding the uniqueness of microbial collagenases in terms of substrate specificity to proteolysis water-insoluble native and water-soluble denatured collagens, and their future perspective in medical sciences, food industry and cosmetic products, introducing novel bacterial collagenases is of a great value^8^.

Proteases that display significant stability at high temperatures while preserving their activity are referred to as thermostable proteins^20^. According to their specific bacterial sources, the optimum temperature differs from one thermostable protease to another and it lies within the range of 40–100 °C^21^. It is important to note that thermotolerancy of the protease is one of the remarkable features, since a great deal of industrial processes are performed at high temperatures in which many of enzymes are not stable^22^.

In the present study we were interested in heterologous expression and biochemical characterization of a novel cysteine protease (1759) from a thermophilic bacterium *Cohnella* sp. A01 (PTCC No 1921). Following the study of expression, structural and biochemical characterization of enzyme was favored in hopes of finding outstanding properties such as considerable thermostability, specificity toward substrate and an increased activity in broad range of different conditions (See Supplementary Fig. S1 online for graphical abstract).

## Results

### Sequence analysis and potential template for homology modeling

**Figure 1:**
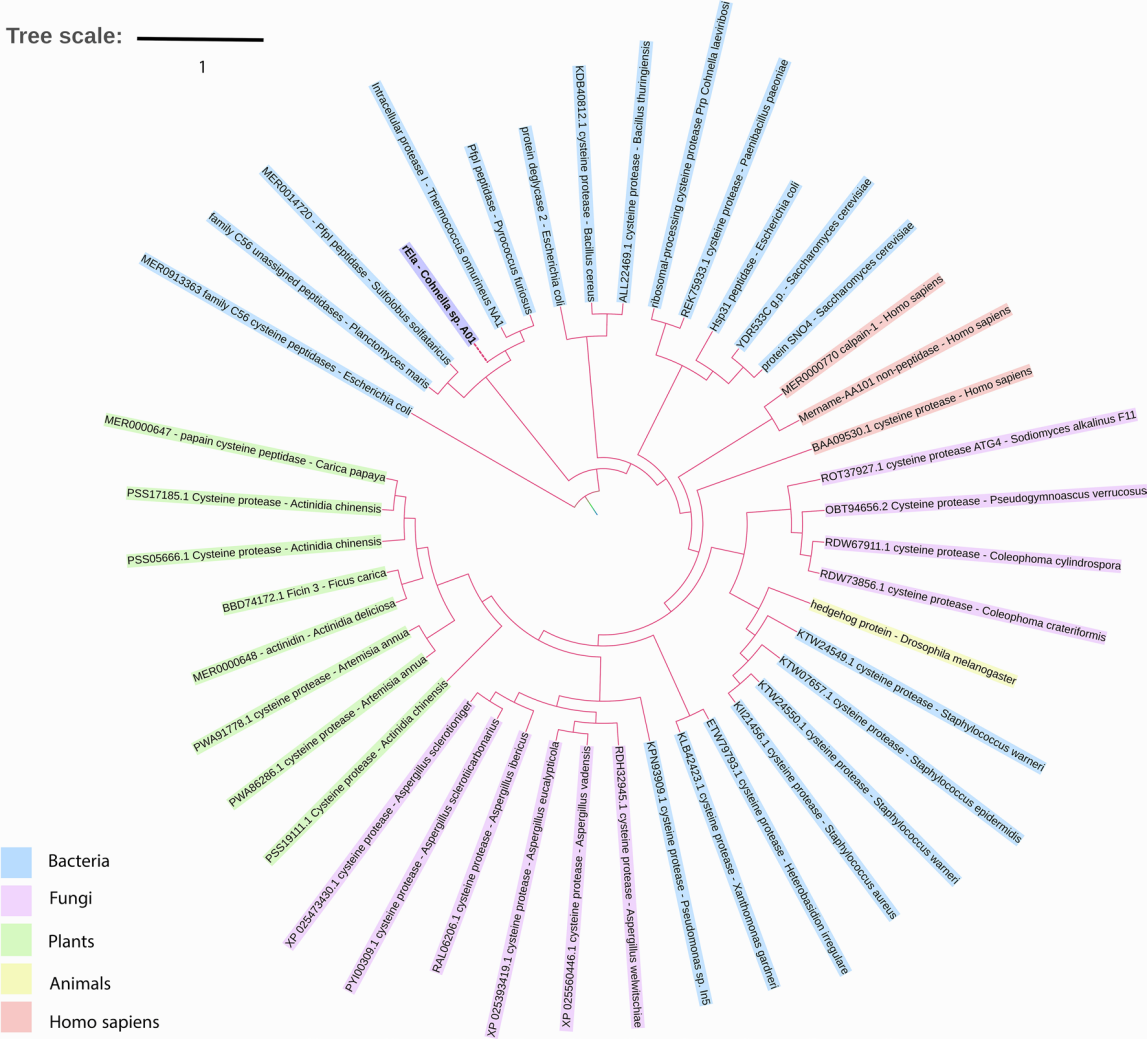
Phylogenetic tree of rEla from *Cohnella* sp.A01 and cysteine proteases from different species. The tree was constructed using ClustalW in MEGA X program based on the alignment of the cysteine protease sequences with high similarities.

rEla cysteine protease sequence translation indicates that it consists of 174 amino acids. SignalP predicted no signal peptide for the enzyme, suggesting the intracellular production of rEla (see Supplementary Fig. S2 online). To analyze the evolutionary relationships, rEla unrooted phylogenetic tree with cysteine proteases from different species was constructed and showed most resemblance with DJ-1/ThiJ/PfpI superfamily of cysteine proteases (Fig. 1). According to MEROPS classification, pfp1 superfamily cysteine proteases belong to C_56_ family with *Pyrococcus furiosus* protease I (PfpI) (a thermostable endopeptidase with significant stability) as the most well- known member^23^.

**Figure 2:**
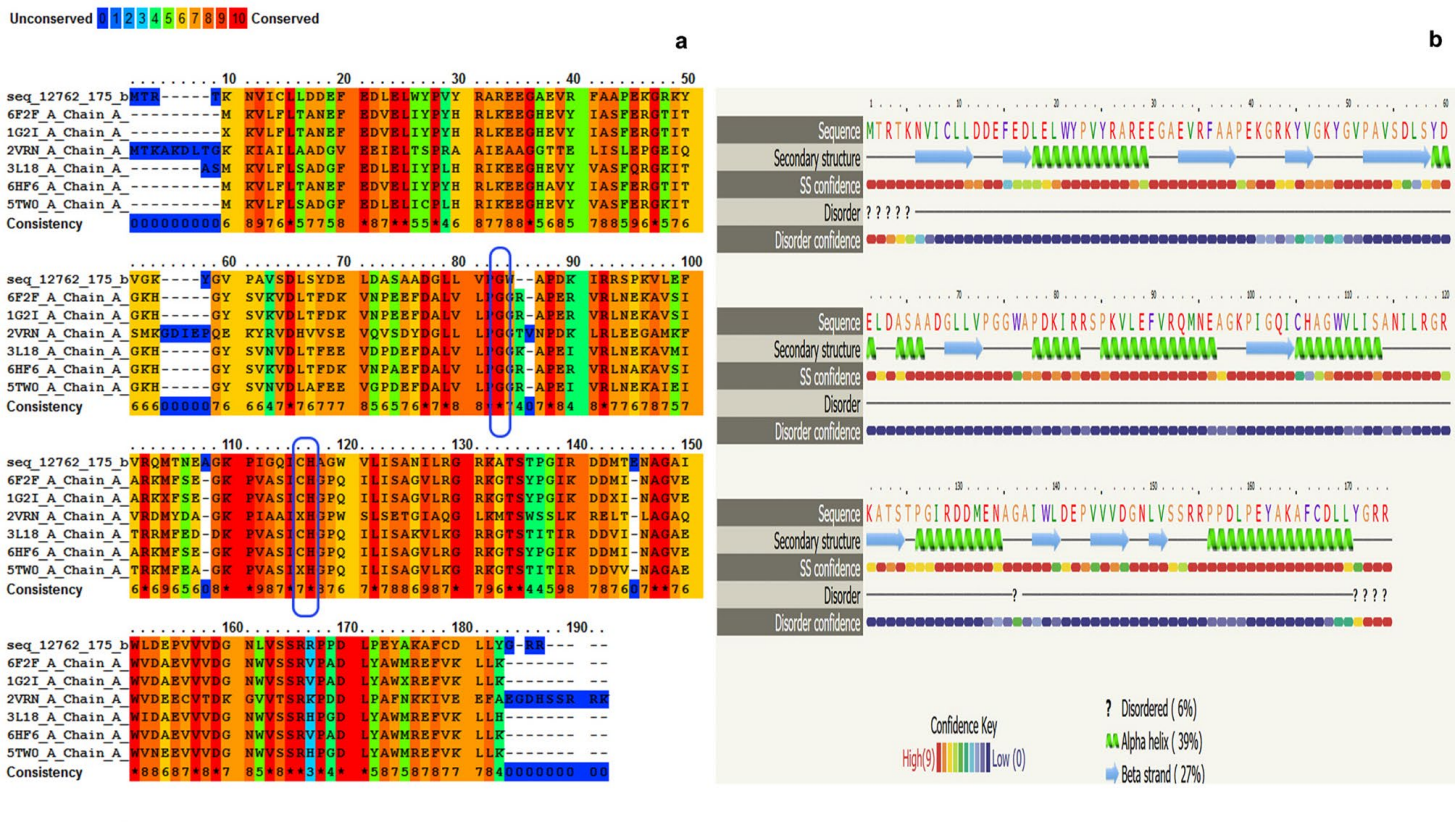
(**a**) Protein sequence alignment of rEla and six closely related sequences. Boxed regions show conserved active site residues. (**b**) Secondary structure of rEla using Phyre2 program. The protease had 39% alpha helix, 28% random coils, 27% beta strand and 6% disordered structures.

In case of finding proteins with similar structures, BLAST of cysteine protease amino acid sequence in the protein data bank (PDB) showed 46.7% identity with protease I from *Pyrococus horikoshii.* To find conserved domains, sequence of rEla cysteine protease was aligned with 6 similar sequences within pfpI superfamily which are characterized by their thermostability and conserved active site residues^24^ (Fig. 2a). It was observed that cysteine, histidine and glycine were three highly conserved amino acids that were considered to be parts of catalytic site of these cysteine proteases.

### Secondary and tertiary structure prediction

**Figure 3:**
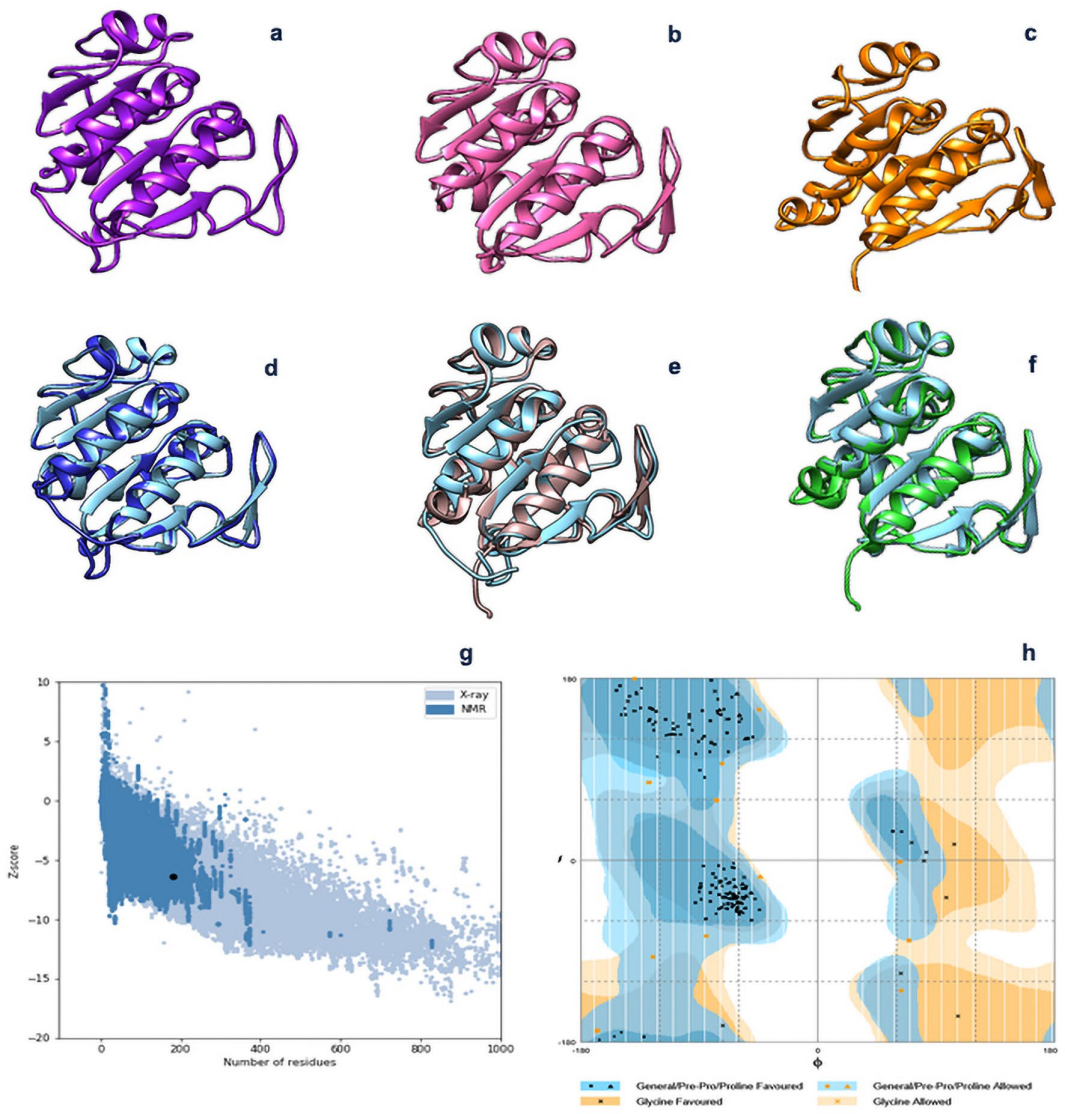
Predicted 3D structure of rEla (**a**) Modeller, (**b**) SWISS-MODEL and (**c**) i-Tasser. (**d**–**f**) superimpose and optimization of (**a**, **b**) structures, (**a**, **c**) models and (**b**, **c**) models with chimera 1.14. (**g**) z-score analysis for homology modelled rEla. The plot exhibited the validation of predicted structure. The black dot shows the similarity of model with X-ray and NMR structures. (**h**) Ramachandran plot revealed that 100% of residues were in favored and allowed regions.

**Figure 4:**
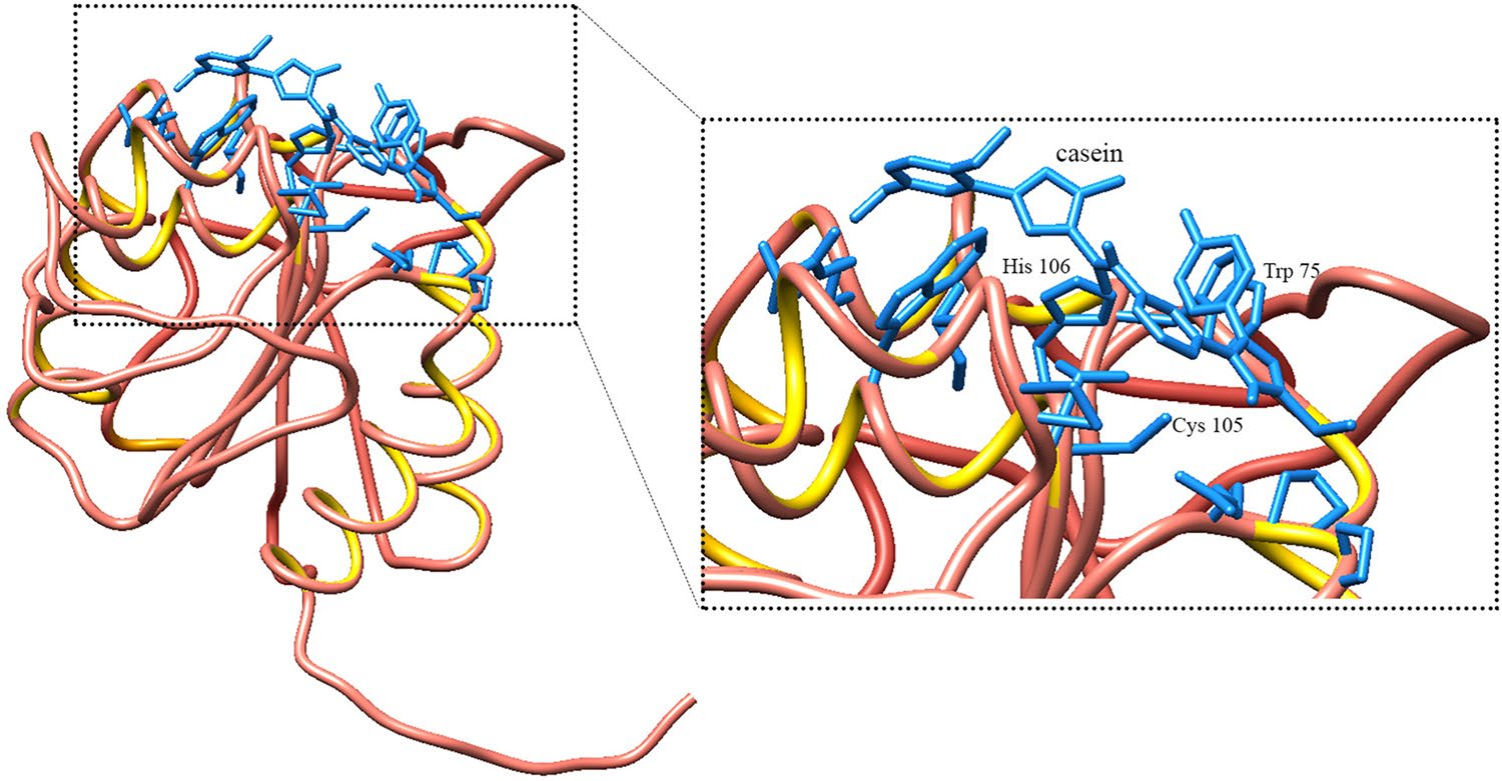
Predicted binding site of cysteine protease in interaction with casein as ligand. Cys 105, His 106, Gly 74 and Trp 75 are predicted catalytic residues.

Secondary structure was predicted by Phyre2 program. Results indicated that the enzyme, consists of 39% alpha helix, 27% beta strands, 28% random coils and 6% disordered structures (Fig. 2b). Tertiary structure was predicted with protease I from *Pyrococus horikoshii* as a template by Modeller 9v7 (Fig. 3a), SWISS model (Fig. 3b) and i-tasser server (Fig. 3c). In order to optimize the generated structures, the predicted models were given to Chimera 1.14 program which corrected the structures from the energy, the chain topology and the hydrogen bonds point of view and improved the physical quality significantly. Following, the structures were superimposed two by two and (Fig. 3d–f) and Root Mean Square Deviation (RMSD) values were 0.385, 0.642 and 0.409 Å respectively, indicating the close resemblance of the generated structures. Finally the 3D structures were assessed by z-score and Ramachandran plots. The best evaluated Z-Score was -6.41 (3D structure built with Modeller 9v7) which shows the similarity of the predicted model to native proteins (Fig. 3g). The results also confirmed the negative energy of nearly most of the residues (see Supplementary Fig. S3 online). Ramachandran plot revealed that 93.3% of residues were in favored and 6.7% in allowed region (Fig. 3h). These supporting data validated the quality of final model. The predicted binding site residues by 3DLigandSite, were Cys 105, His 106, Gly 74 and Trp 75 (Fig. 4). The modeled protease structure was used for further docking studies.

### rEla-ligand docking, and molecular dynamics

**Figure 5:**
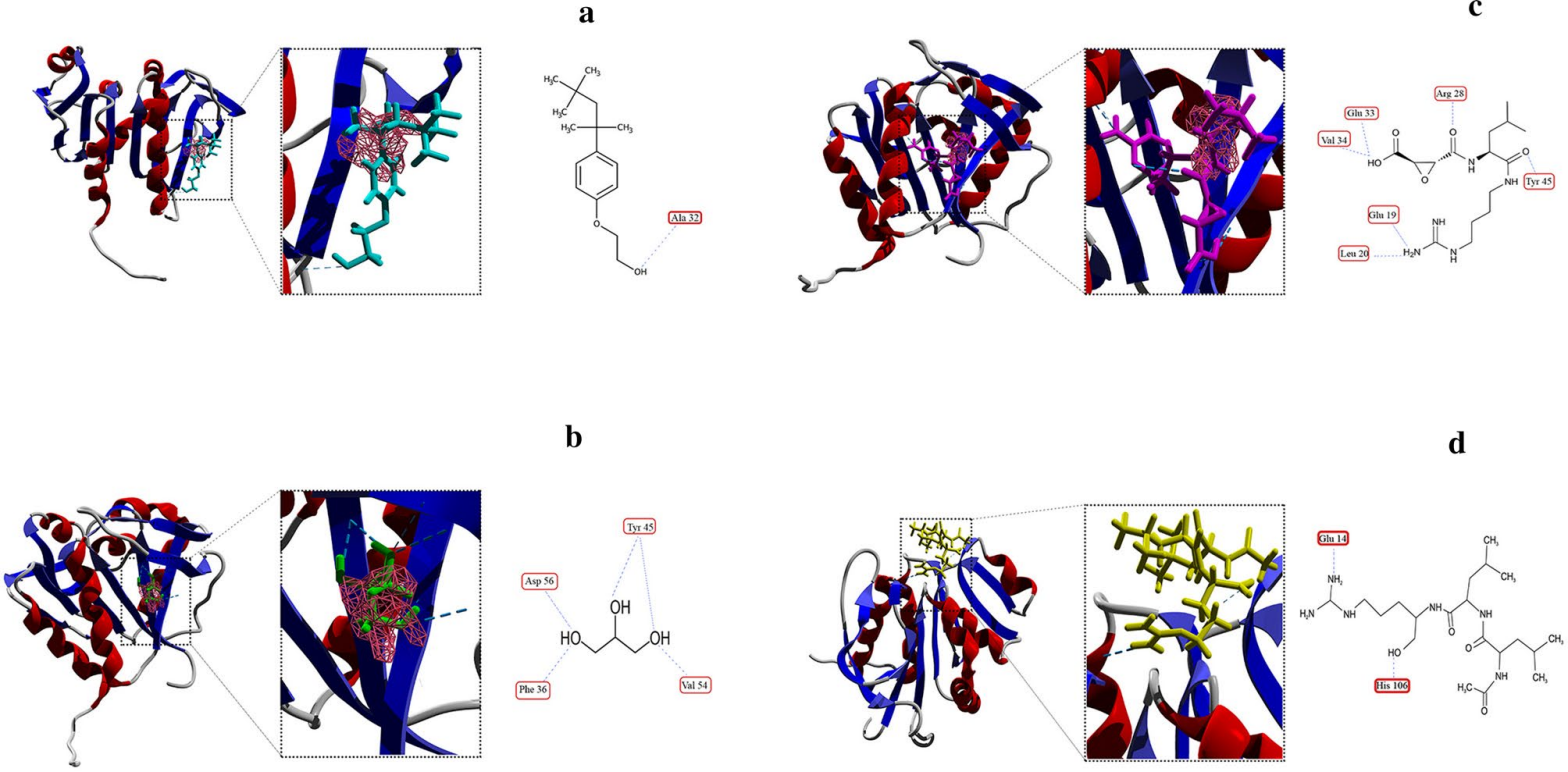
(**a**) Docking of rEla and Triton x-100. Triton x-100 is a surfactant with an increasing impact on protease activity. 2D display shows interaction between Ala 32 of rEla with oxygen atom of Triton x-100. (**b**) Docking of glycerol as a ligand and rEla. Glycerol interacts with allosteric cavity via 5 hydrogen bonds as 2D structure represents. Molecular docking of two cysteine protease specific inhibitors E.64 (**c**) and Leupeptin (**d**). E.64 seems to inhibit the enzyme by 6 hydrogen bond interactions with allosteric cavity and changing the protease active conformation. Unlike E.64, Leupeptin is a competitive inhibitor which interacts with active site via 2 hydrogen bonds with His-106 and Glu-14.

**Figure 6:**
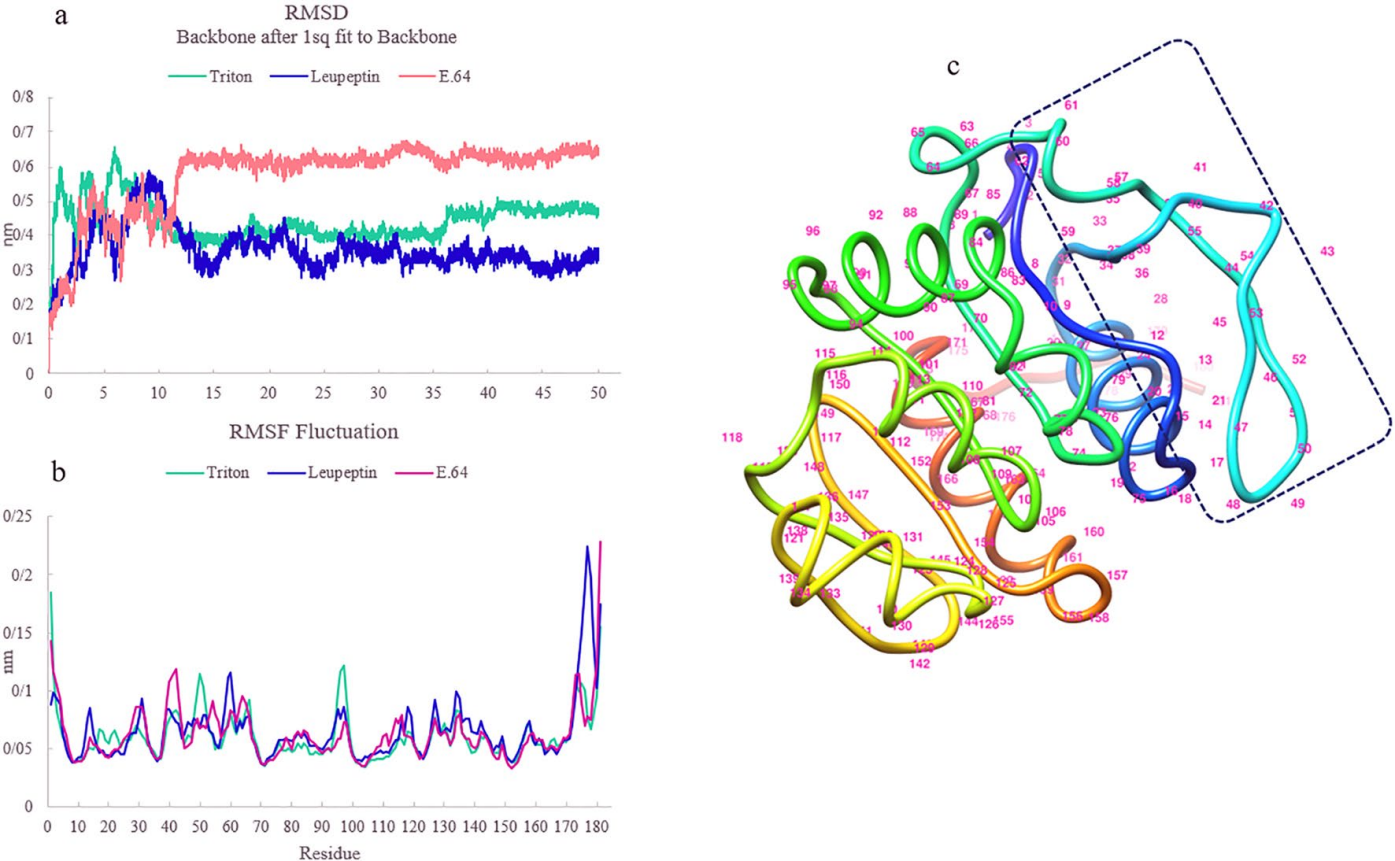
MD simulation of rEla cysteine protease in complex with Triton X-100, E.64 and Leupeptin. (**a**) RMSD graph, (**b**) RMSF graph and (**c**) The structure of flexible loop effective on activation/deactivation of rEla. The loop is located between two β-sheets and consists of residues from 39 to 63.

To comprehend the proteolytic activity of the predicted protease model, the cysteine protease was docked with 4 ligands with supposedly increasing and inhibitory effects on activity. The docking results overall exhibited a second binding site which seemed to be allosteric site of rEla cysteine protease. The docking interaction of enzyme with Triton X-100 was predicted through a hydrogen bond between oxygen atom of Triton X-100 and Ala 32 residue with a distance of 2.63 Å and docking score of − 75.52 kJ/mol (Fig. 5a). Molecular docking of the allosteric cavity and glycerol showed 5 hydrogen bonds with Phe 36, Tyr 45 (2 bonds), Asp 56 and Val 54 in distance of 3.12, 3.01, 2.93, 2.97 and 3.21 Å respectively (Fig. 5b). Figure 5c and d shows the docking results of the rEla predicted model with 2 cysteine protease specific inhibitors E.64 (c) and Leupeptin (d). E.64 interacts with allosteric cavity (supported by Glu 19, leu 20, Arg 28, Glu 33, Val 34 and Tyr 45) with dock score of − 107.5 kJ/mol but Leupeptin interacts with His 106 residue of active site and Glu 14 with dock score of − 107.01 kJ/mol. The docked complexes were then simulated for a period of 50 ns and the interactions were maintained during 50 ns MD simulations. MD results indicated that after 50 ns of molecular dynamic simulation, the structure was stable and the bonds were preserved (Fig. 6). The RMSD result shows stable behavior (Fig. 6a). E.64 has the highest value indicating the highest conformational changes in protein structure. RMS fluctuation plot (Fig. 6b) overall shows larger fluctuations for the ends of the protein and small fluctuations for the rigid structural elements. The plots indicate that Triton X-100, Leupeptin and E.64 interactions with protein shows considerable fluctuations in different parts of a certain loop consisting of residues from 39 to 61. Figure 6c highlights the aforementioned loop, responsible for the flexibility changes in the enzyme structure.

### Structural model of rEla substrate-binding site

**Figure 7:**
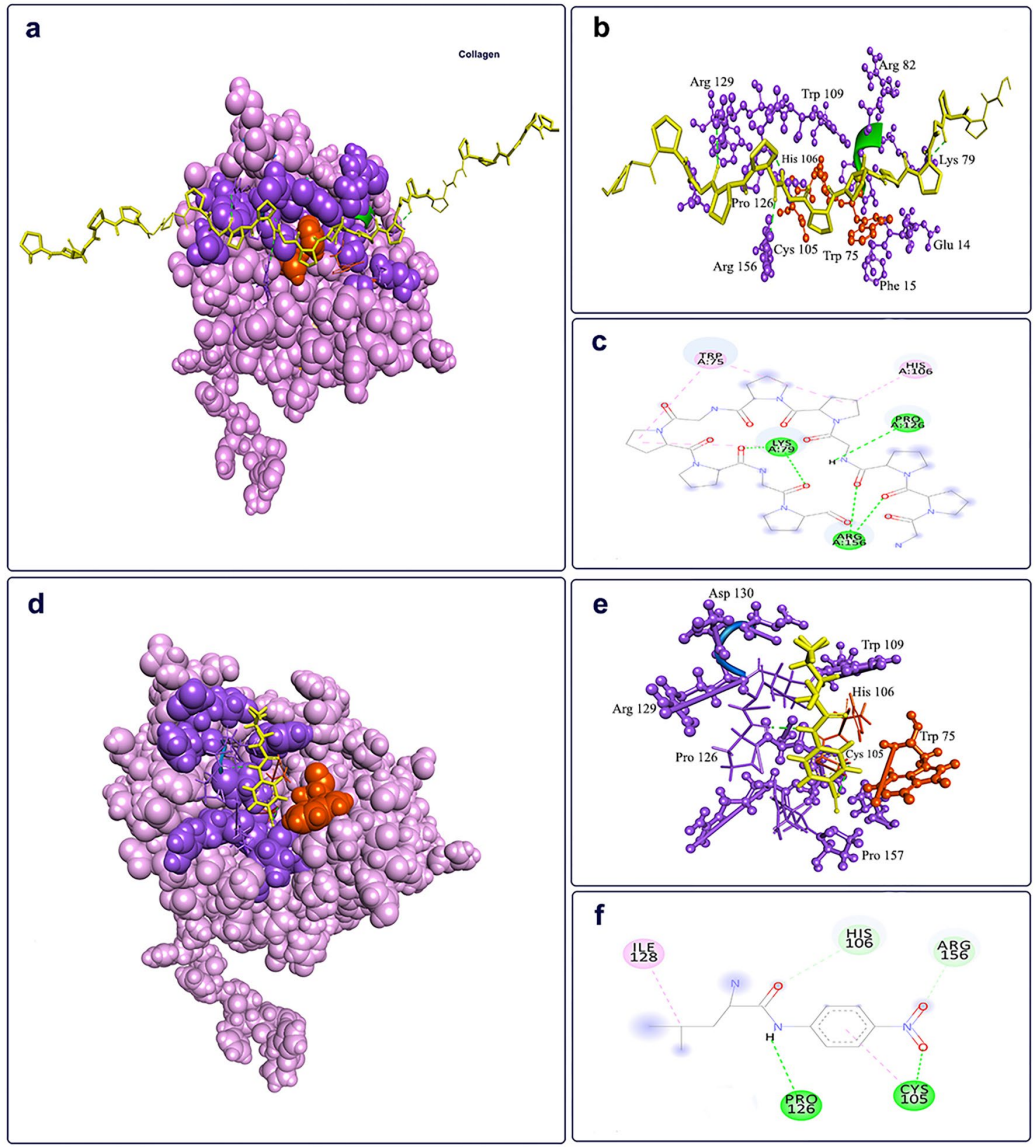
The active site and substrate docking of rEla. The active site residues are shown in purple and catalytic residues are shown in orange. The yellow color shows the substrates. (**a**) Docked pose for rEla in complexed with Collagen, (**b**) Active site pocket of rEla with collagen as the substrate, (**c**) 2D interactions of rEla binding site with collagen. Collagen interacts with rEla through 4 hydrogen bonds with Lys 79, Pro 126 and Arg 155. Hydrogen bonds are shown in green and hydrophobic interactions are pink. (**d**) Docked pose of rEla in complexed with l.leucine.p.nitroaniline, (**e**) Active site amino acid residues from docking with l.leucine.p.nitroaniline and (**f**) 2D interactions of rEla amino acids with l.leucine.p.nitroaniline. The enzyme binds to l.leucine.p.nitroaniline through Cys 105 and Pro 126.

To investigate the binding site and the interactions with different substrates, molecular docking of rEla with 2 substrates, collagen (as the specific substrate that rEla showed high activity toward it) and l.leucine.p.nitroaniline (rEla had relatively low activity against it) was carried out (Fig. 7a,d). As Fig. 7b shows, Glu 14, Phe 15, Trp 75, Lys 79, Arg 82, His 106, Trp 109, Pro 126, Arg 129 and Arg 156 are important rEla residues that form substrate active site. While Trp 75, Cys 105, Trp 109, Pro 126, Arg 129, Arg 155 are the main amino acids in l.leucine.p.nitroaniline interactions with rEla (Fig. 7e). The catalytic amino acids of rEla active site as mentioned before are Cys 105, His 106 and Trp 75. As the docking results display, the active site residues overlap but collagen interaction with enzyme seems to involve more amino acids through 4 hydrogen bonds with Lys 79, Pro 126 and Arg 155 (Fig. 7c). l.leucine.p.nitroaniline binds with Cys 105 and Pro 126 (Fig. 7f).

### Gene cloning, heterologous expression and purification of *Cohnella* sp. A01 rEla cysteine protease (1759)

**Figure 8:**
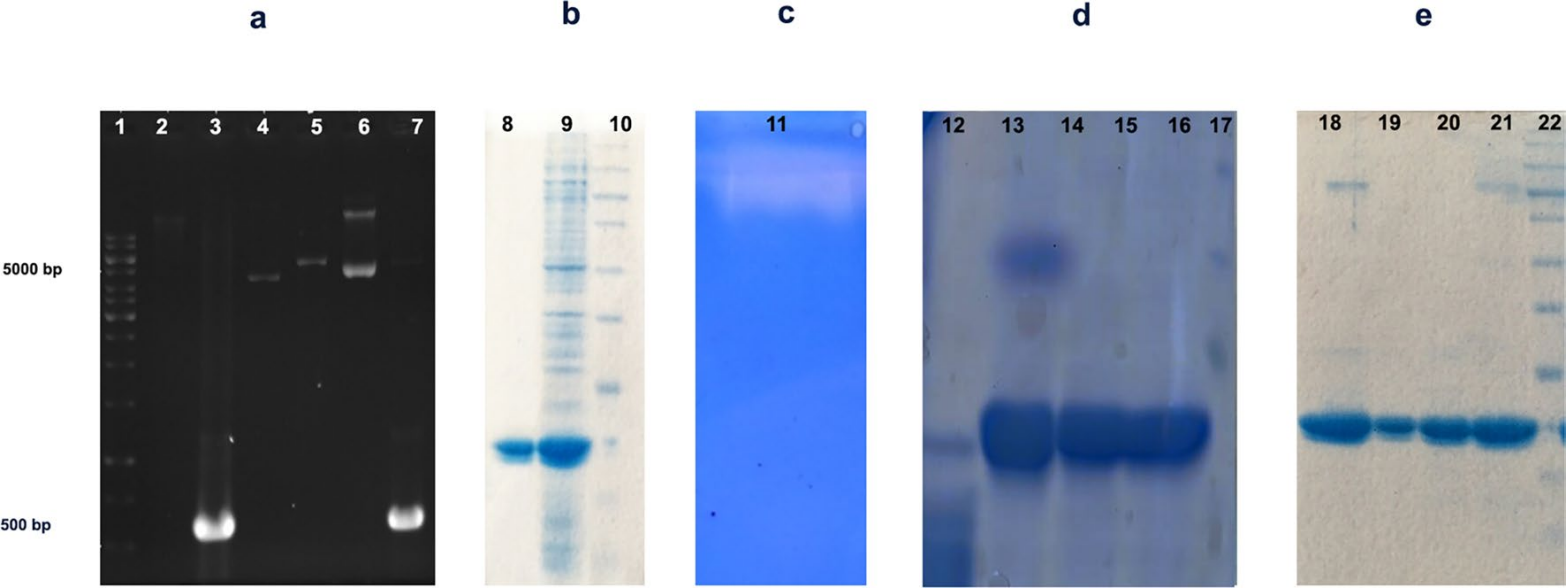
(**a**) Agarose gel of cloned gen. lane 1: DNA ladder, lane 2: extracted gene from *Cohnella* sp. A1, lane 3: PCR product, lane 4: pET26-b, lane 5: non-recombinant plasmid, lane 6: recombinant plasmid, lane 7: colony PCR, (**b**) SDS-PAGE analysis of expressed rEla gene. Lane9: purified rEla. Lane 10: total cellular protein expressed of *E. coli* BL21.Lane 11: protein molecular mass marker. (**c**) Casein zymography of rEla (lane 11). (**d**) rEla resistance against some proteases. Lane 12: proteinase K, lane 13: Trypsin, lane 14–16: rEla, lane 17: protein molecular mass marker. (**e**) SDS-PAGE analysis of rEla thermal stability after 100 days. Lane 18: enzyme stored at 4 °C, lane 19: − 20 °C with 20% glycerol, lane 20: − 20 °C and lane 21: 25 °C. Lane 22: protein molecular mass marker. Full-length gels are presented in Supplementary Fig. S4 online.

Cloning of *Cohnella* sp. A01 rEla gene into pet-26b(+) vector was successfully carried out and is shown in Fig. 8a.The sequence analysis indicated that the gene encoded a 19.38 kDa protein with 174 amino acids. The heterologous expression of intended protease was confirmed through SDS-PAGE analysis. The enzyme was successfully purified by Nickel-Sepharose resin affinity chromatography during which, rEla was purified with 88% yield (Fig. 8b). For full-length gel see Supplementary Fig. S4 online.

Casein zymography is an electrophoretic method based on NATIVE-PAGE to detect protease activity in gel by using casein as a substrate^25^. Therefore the catalytic activity of rEla was investigated and the clear zone shown in Fig. 8c corroborated the hydrolytic activity. Full-length gel is presented in Supplementary Fig. S4 online.

### rEla resistance against proteases

Proteinase K and trypsin digestive effect on rEla was carried out (For full-length gel see Supplementary Fig. S4 online) and the SDS-PAGE data indicates that the cysteine protease was for the most part hydrolyzed by proteinase K, but remained approximately intact by trypsin (Fig. 8d).

### Effects of temperature and pH on enzyme activity and stability

**Figure 9:**
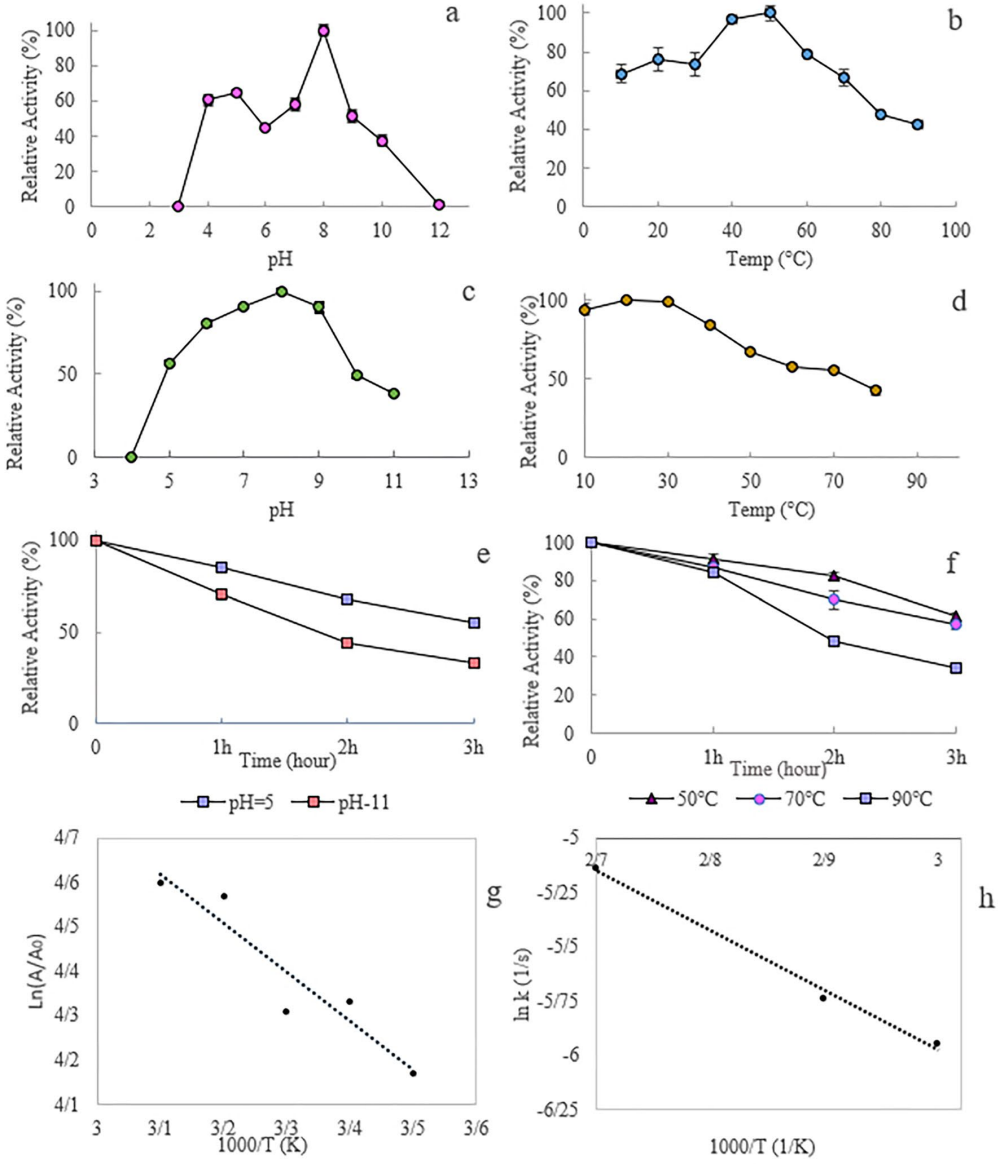
The effect of temperature and pH on rEla. (**a**) pH profile, the optimum pH for catalytic activity of the enzyme was 8. (**b**) Temperature profile, the optimum temperature of the enzyme activity was 50 °C. rEla showed more than 70% of residual activity at the temperature range of 20–60 °C (**c**) pH stability, (**d**) temperature stability, (**e**) pH stability at pH 5 and 11 in different times, (**f**) temperature stability at 50, 70 and 90 °C at different times. (**g**) Arrhenius plot for E_a_^*^ and (**h**) E_a_^#^.

Figure 9 shows the effect of temperature and pH on rEla activity and stability. The Enzyme revealed high stability in pH ranges from 5 to 9 and showed highest activity at alkaline pH 8 (Fig. 9a). rEla optimum temperature was 50 °C (Fig. 9b) and enzyme remarkably exhibited over 70% activity at temperature range of 20–60 °C. After 90 min incubation the relative activities of rEla was more than 80% in the pH range of 6–9 (Fig. 9c) and enzyme preserved more than 70% of its residual activity at10–70 °C (Fig. 9d). In another stability experiment rEla showed 67% and 44% activity after 2 h incubation at pH 5 and 11, respectively (Fig. 9e). Thermostability studies revealed that the enzyme preserved about 60% activity after 3 h incubation at 50 °C (Fig. 9f) and it was up to 55% stable after 2 h incubation at 70 °C. Even after incubating the cysteine protease at 90 °C for 2 h, rEla remained about 50% active.

### Determination of kinetic and thermodynamic parameters

Michaelis–Menten plot was used to determine kinetic constants (see Supplementary Fig. S5 online). K_m_, V_max_ and k_cat_ values of the cysteine protease with casein as substrate were obtained 21.93 mM and, 8 U/ml and 8.6 s^−1^, respectively. To compare the kinetic parameters of different cysteine proteases with rEla see supplementary Table S1 online.

The activation energy was calculated 9.145 kJ/mol by Arrhenius plot (Fig. 9g) which is the amount of energy that must be provided for rEla to form E–S complex. ΔG^*^, ΔH^*^ and ΔS^*^ at optimum temperature were 79, 6.39 kJ/mol and − 0.22 kJ/molK, respectively.

To measure the thermodynamic parameters of enzyme irreversible thermo-inactivation, thermal inactivation plot at 50, 70 and 90 °C was drawn (see Supplementary Fig. S5 online). k_in_ was obtained for each of the above temperatures and as shown in Table 1, k_in_ increases gently as temperature goes up. The Arrhenius plot was then designed for mentioned reaction (Fig. 9h). Activation energy E_a_^#^ value was calculated 22.78 kJ/mol. △G^#^, enthalpy △H^#^ and entropy (△S^#^) were 95.3 kJ/mol, 22.1 kJ/mol and − 0.226 kJ/molK at optimum temperature. Table 1 displays negative amounts of entropy, suggesting the negligible disorder and therefore reasonable thermotolerance of rEla.

**Table 1:** Thermodynamic parameters for irreversible thermal inactivation of rEla.

Temperature ℃	K^in^	E_a_ (kJ/mol)	ΔH^#^ (kJ/mol)	ΔG^#^ (kJ/mol)	ΔS^#^ kJ/molK	t_1/2_ min
50	0.0026	22.7800	22.1000	95.3000	− 0.2260	265
70	0.0032		19.9300	100.7800	− 0.2350	215
90	0.0062		19.7600	104.8300	− 0.2340	111

### rEla long-term storage

**Figure 10:**
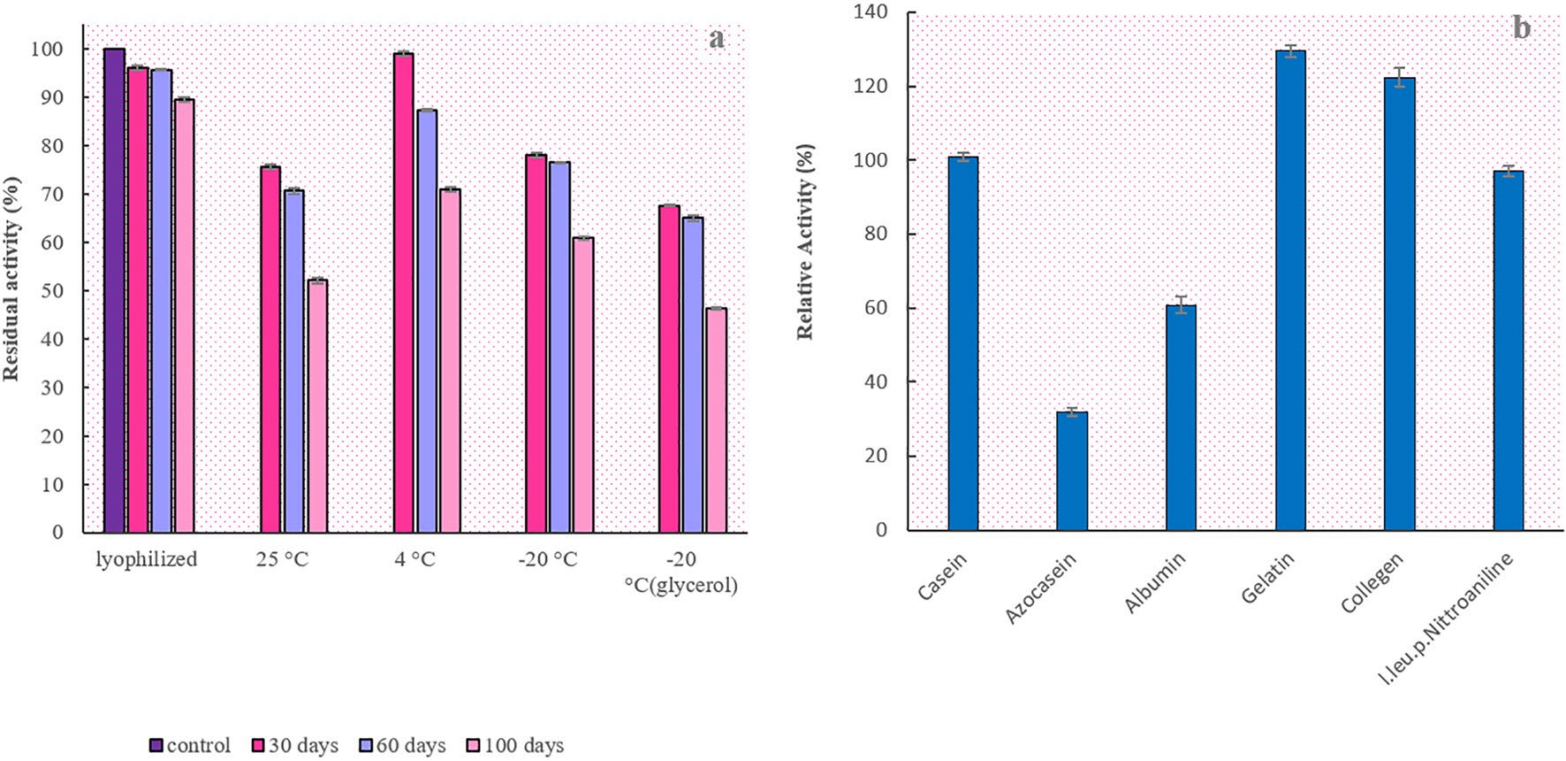
(**a**) Relative activity of the enzyme during 100 days of storage. rEla showed remarkable stability and the enzyme half-life at room temperature was about 100 days which makes it an excellent candidate for different industrial applications (**b**) rEla substrates specificity. The enzyme activity in presence of gelatin and collagen was 130% and 120% respectively.

In order to assess rEla long-term stability, the enzyme was kept in 25, 4, and − 20 °C for 100 days. Along other samples lyophilized enzyme was also incubated at 4 °C. The SDS-PAGE analysis was run after 100 days to observe alterations in enzyme electrophoretic pattern (Fig. 8e) and no noticeable changes were observed. The activity was examined at day 30, 60 and 100 as shown in Fig. 10a. All of the enzyme samples preserved more than 60% activity after 2 months. The rEla activity results after 100 days indicates that lyophilized protease exhibited the best stability by retaining 89% activity and the enzyme kept at -20℃ with glycerol, lost its residual activity more than other samples and was only 46% active.

### Substrates specificity

The Effects of different substrates on enzyme activity were assessed using conventional protease substrates including casein, azocasein, albumen, gelatin, collagen and l.leucine.p.nitroaniline. The enzyme showed the highest activity toward gelatin and collagen with the relative activity of 130 and 120%, respectively and exhibited the least activity against azocaseine with the relative activity of 32% (Fig. 10b).

### Effects of organic solvents, surfactants and metal ions on enzyme activity

**Figure 11:**
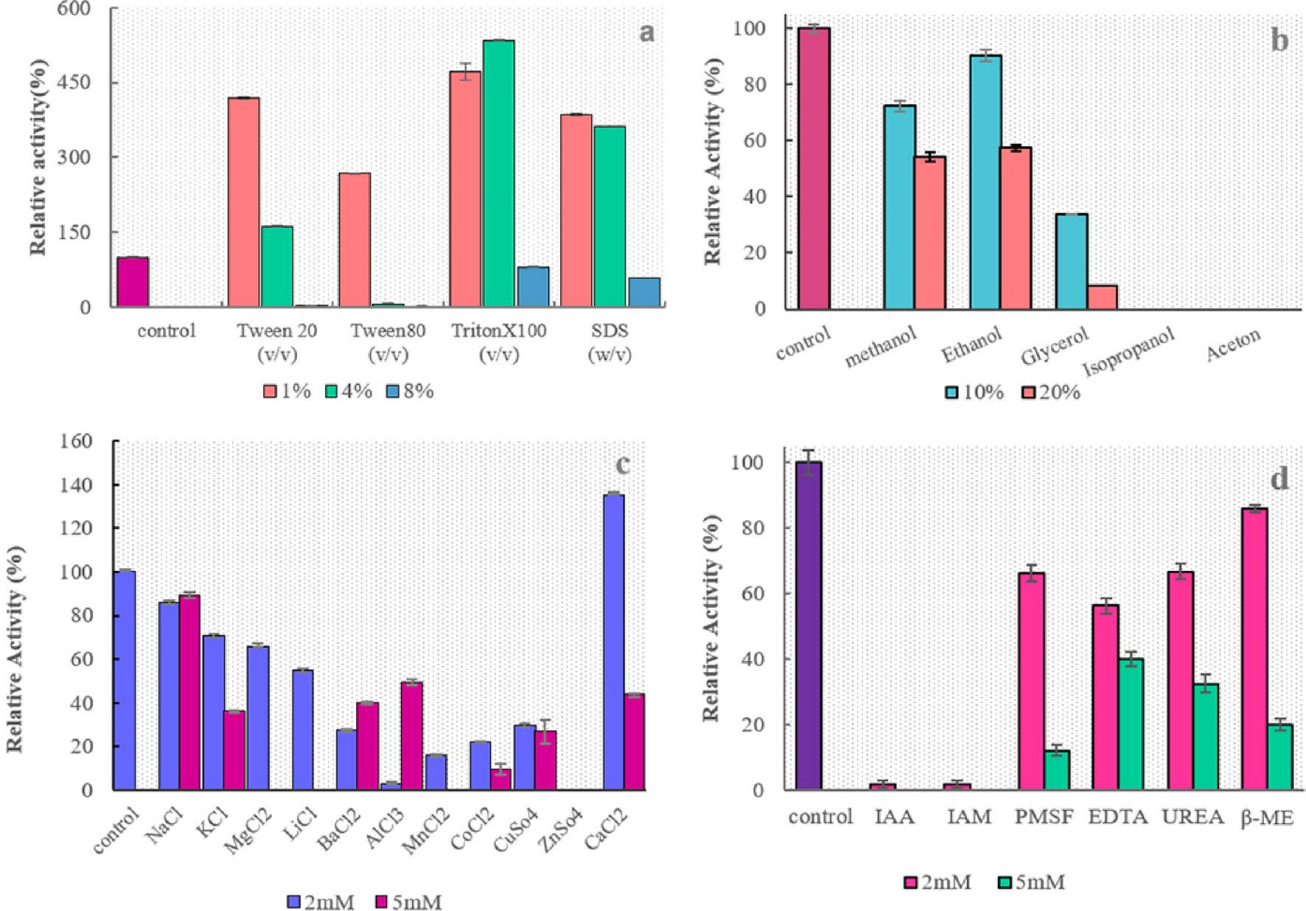
The effect of (**a**) surfactants, (**b**) organic solvents, (**c**) metal ions and (**d**) inhibitors on rEla relative activity.

The influence of organic solvents, surfactants and metal ions (2 and 5 mM), on the proteolytic activity of purified rEla was investigated at optimum activity conditions (Fig. 11). The effect of several surfactants at the concentration of 1, 4 and 8% (w/v) on enzyme activity was studied (Fig. 11a). The Proteolytic activity was not significantly changed in the presence of 10% ethanol and 90% of activity was preserved in comparison to the control, although adding 20% ethanol leads to reduction of enzyme activity to 57% (Fig. 11b). Exposing the enzyme to isopropanol and acetone ceased the activity. The enzyme retained more than 70% of its activity following exposure to 10% methanol. 10% glycerol decreased the enzyme activity to less than 40%.

The observations showed increase in enzyme activity at 1% concentration of Tween 20, Tween 80, Triton X-100 and SDS up to 419,117, 472 and 386% respectively. Triton X-100 at concentration of 4% had the maximum influence and increased proteolytic activity to 490%.

Figure 11c shows that enzyme activity was increased in the presence of Ca^2+^ ions with final concentration of 2 mM. The enzyme maintained less than 60% of its maximum activity after treatment with 5 mM of KCl, AlCl_3_, BaCl_2_, CaCl_2_ and 2 mM concentration of LiCl. The addition of Na^+^ and K^+^ decreased proteolytic activity gradually so the relative activity of purified enzyme was approximately about 80% and 70%, respectively. Moreover, at 5 mM ZnSO_4_, Li^+^ and Mg^2+^ enzyme activity was totally lost. Supplementary Table S2 shows effect of different metal ions on some cysteine proteases.

### Effect of inhibitors on the enzyme activity

The inhibitory impacts of different chemical compounds on rEla activity are shown in Fig. 11d. IAA and IAM completely ceased the enzyme activity as they are cysteine protease inhibitors, while PMSF (2 mM) failed and the protease maintained more than 60% of its residual activity. EDTA, Urea and β-ME at 5 mM concentration, lowered activity to less than 25%.

**Figure 12:**
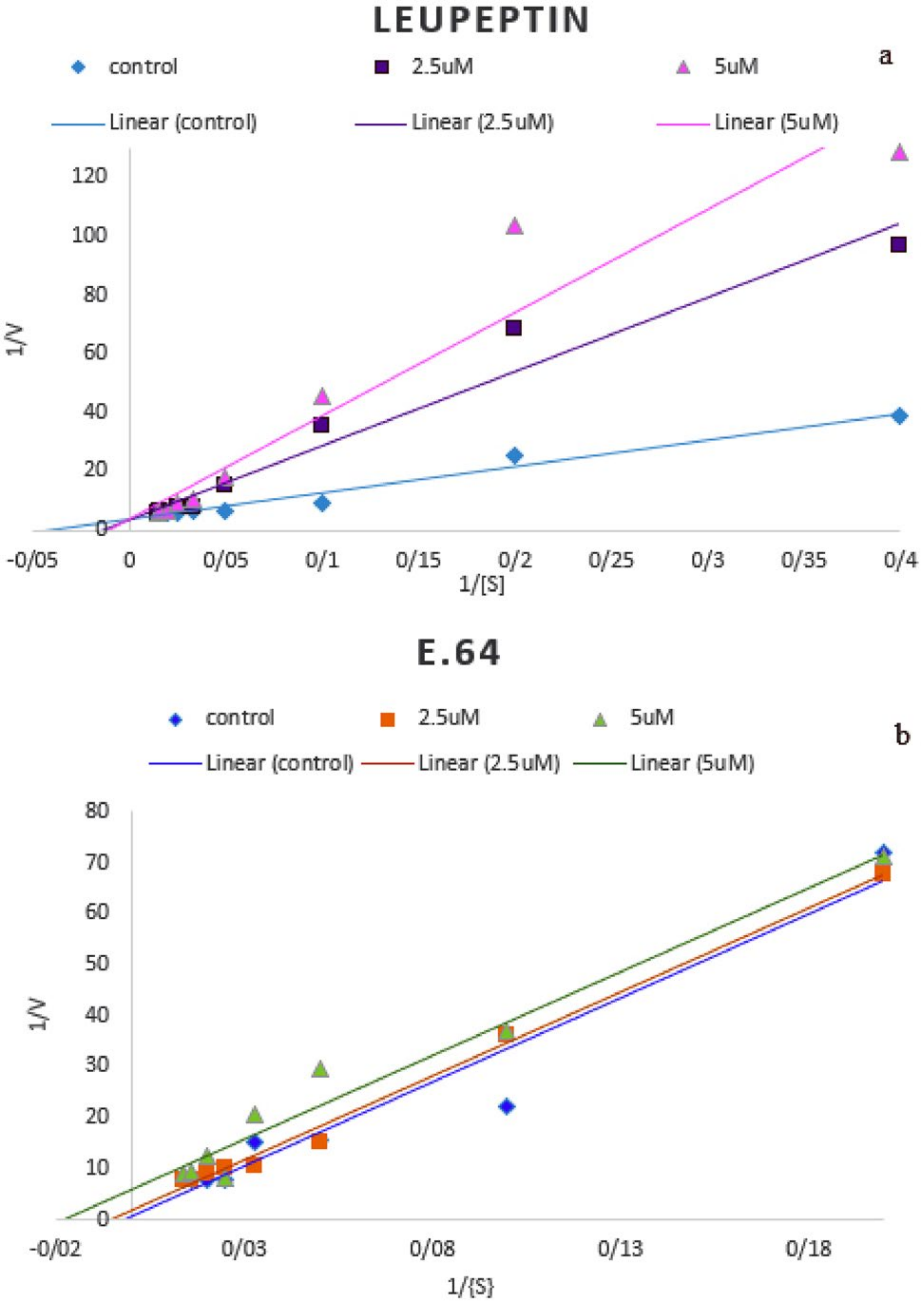
The study of enzyme activity in presence of specific inhibitors (**a**) Leupeptin (**b**) E.64. Leupeptin is a competitive inhibitor which changes k_m_ and E.64 is a uncompetitive inhibitor and changes both k_m_ and v_max_ of the enzyme.

In order to confirm the type of inhibitor behavior, the enzyme activity was measured in the presence of two specific inhibitors, Leupeptin and E.64 at 2.5, 5, and 10 mM (see Supplementary Fig. S6 online) which belong to the group of small inhibitors^26^. leupeptin competitively inhibited the enzyme activity while E.64 inhibitory impact was uncompetitive (Fig. 12).

## Discussion

Bacterial proteases are one of the most important and well-studied parts of hydrolytic enzymes with numerous industrial and medical applications^1^. The utilization of recombinant enzymes has privilege over native proteins, as the amount of purified protein increases significantly and enzymatic properties such as stability are commonly improved^27^.

Here, in this study we heterologously expressed the cysteine protease gene from a novel thermophilic bacterium, *Cohnella* sp. A01. In silico analysis indicate that rEla is an intracellular protease. It has low sequence similarity to other reported proteases with the highest identity of 46.70%, and is thus a novel alkaline cysteine protease. Phylogenetic analysis of rEla gene sequence indicates that it is a cysteine protease from DJ-1/ThiJ/PfpI superfamily. Despite growing fast and having representative in most of the organisms, only few members of this superfamily have been characterized biochemically^28^. Sequence alignment of rEla and homologous gene sequences exhibits the conserved catalytic triad His_105_, Cys_106_ and Gly_74_. The activity of all cysteine proteases depends on the catalytic dyad consisting of cysteine and histidine. The order of Cys and His (Cys–His or His–Cys) residues differs among the families^29^.

Protease I from *Pyrococus horikoshii* was considered as the most similar template to build 3D structure. The models constructed with Modeller 9v7, SWISS-model and I_TASSER were superimposed in chimera 1.14 and very low RMSD values verified the close similarity of all generated models. The Ramachandran plot, without any outlier residues, validated the modeled structure. Further verification was carried out using ProSA. The calculated Z-score (− 6.41) displayed a compatible value with native proteins and an acceptable negative balance for the potential energy of the predicted model^30^.

Due to their flexible binding cavity, allosteric sites allow ligands binding to the allosteric pocket in a way that forms the best conformation (low energy conformations as zymogen state) to inhibit the enzyme, therefore in their presence the residual activity of the enzyme would be very low to zero^31^. Docking and MD simulation conclusions indicate that rEla has an allosteric site capable of having interactions with small molecules, resulting in increase or cease of the catalytic activity. E.64 is a cysteine protease inhibitor. The results exhibit the interaction between E.64 and allosteric site of rEla, causing inactivation of the enzyme via stable conformational changes. RMSF result shows fluctuation in residues from 39 to 43 leading to more rigidity of the enzyme. Triton X-100 is a nonionic detergent and it was shown that its presence has a great impact on increasing the protease activity. The results indicate that Triton X-100 interacts with Ala 32 residue of the allosteric site through hydrogen bond and induces the fluctuation changes in residues from 49 to 52 locating in a big loop near active site of the enzyme. Since all RMSF results emphasis on fluctuation changes of residues lying in this area, this particular loop seems to have a significant effect on rEla flexibility and rigidity. Leupeptin docking suggests that it is a competitive inhibitor. It interacts with active site of rEla and prevents the enzyme–substrate complex formation. Therefore, MD simulation results confirm docking outcomes and show maintenance of the interactions after 50 ns.

Docking results indicate that the substrate binding pocket of rEla consists of more than 10 amino acids and is mostly identical for both substrates. Collagen and l.leucine.p.nitroaniline both interact with Pro 126 but have different spatial orientations with respect to the main residues of the active site. This might lead to the higher activity and more specificity of rEla toward collagen. On the other hand, as Fig. 7f displays, l.leucine.p.nitroaniline bonds with Cys 105 which is one of the catalytic residues in rEla active site. Therefore, the main catalyzing reaction is somehow blocked as l.leucine.p.nitroaniline binds to the active site. This might lead to the low activity of rEla toward l.leucine.p.nitroaniline as the results confirm laboratory data outcomes.

The molecular mass of the purified protease was estimated 19.38 kDa. Most of the characterized cysteine proteases are small proteins with molecular masses in the range of 20 to 40 kDa^2,32–42^.

Proteinase K is a nonspecific endopeptidase which doesn’t need specific sites on proteins to digest them^43,44^. On the other hand trypsin as a specific protease, cleaves only the C-terminal of lysine and arginine residues^45^. Thus, proteinase K is able to break down rEla more freely. On the other hand, rEla has 21 specific cleavage sites for trypsin to hydrolyze it. Only 8 of these cleavage sites are on the surface of rEla. Therefore, in comparison with proteinase K, trypsin might be very limited. In addition, due to rEla high rigidity some of these 8 cleavage sites might be inaccessible^46^.

Studies on the thermodynamic stability of proteases brings us closer to the factors that ascertain the enzyme stability^47^, therefore in order to comprehend rEla’s behavior under various physiological conditions, the thermodynamic parameters of the protease were calculated^48^. As we have shown in this study, rEla has structural stability and remains active at high temperatures.

The low k_in_ at 50 °C (0.0026) and notable high t_1/2_ (256 min) confirmed the thermostable structure of the enzyme. The Arrhenius plot for irreversible thermos-inactivation was linear. Positive amount of △G^#^ value indicates that the inactivation of enzyme is not spontaneous. The high Ea for inactivation means high amount of energy is needed for denaturation of rEla and therefore the enzyme is stable with temperature.

The rEla cysteine protease exhibited maximum activity at 50 °C similar to the cysteine protease extracted from *Cissus quadrangularis*^39^*.* Cysteine proteases isolated from *Zingiber montanum* rhizome, *Ficus johannis*, *Calotropis procera*, *Z. officinale*, *Ficus johannis*, exhibited optimum temperature at 60 °C which are all in the optimum temperature range of rEla^4,32,39,41^. The enzyme activity was stable with temperature at the range of 10 to 70 °C and it displayed thermostable features and maintained up to 60% of its proteolytic activity after incubation at 50 °C for 3 h. In a study held by K. Jamir and K. Seshagirirao cysteine protease isolated from *Zingiber montanum* rhizome 50% inactivation was observed by incubating the protein for 45 min at 40 °C and the enzyme was completely inactivated at 70 °C^32^ while, rEla retained about 40% of its maximum activity after 3 h incubation at 90 °C. High thermal stability is an important aspect of proteases required for using them in different applications. Increasing temperatures might cause destruction of non-covalent bonds within a folded protein, which leads to unfolding the protein^49^. Despite the great universal demand for enzymes that can tolerate extreme environmental conditions, the total number of them are very limited^50^. On the other hand, these proteases are capable of keeping the contamination problems to a minimum which would be very helpful in producing and applying them in large quantities^51^.

As we showed rEla was stable in pH range 5–9. Although enzyme highest activity was observed at alkaline range at pH 8, it showed better stability in acidic range. To the best of our knowledge aside from the *Zingiber montanum* extracted cysteine protease with optimum pH 9*,* the enzyme had the highest optimum pH among previously studied cysteine proteases. The enzyme’s remaining activity after 3 h treatment was 60% at pH 5, while it was less than 40% at pH 11. In the study held by Kizukala Jamir, Kottapalli Seshagirirao, ZCPG cysteine protease from *Zingiber montanum* rhizome was active in the pH range from 6.0 to 11.0 and preserved more than 61% and 81% of its activity at pH 7.0 and 10.0 respectively^32^. Thus rEla protease is considered alkaline protease as it can perform high activity at alkaline pH, e.g., pH 9. Alkaline proteases optimum temperature is usually around 60 °C and they have an extensive substrate specificity. These properties are essential for proteases applications in detergent industry^5^. Molecular weight, optimum temperature and pH and pI of some cysteine proteases from different sources are summarized in Table 2.

**Table 2:** Molecular weight, optimum temperature, pH and pI of some of the well-studied cysteine protease.

Name	Source	Weight kDa	Optimum temperature °C	Optimum pH	pI	Reference
rEla	*Cohnella* sp. A01	19.38	50	8	5.33	This study
Zingiber montanum cp	*Zingiber montanum rhizome*	48 k	60	9	4.80 and 5.10	^32^
Ficin	*Ficus johannis*	25	60	7		^35^
EhCP6	*Entamoeba histolytica*	40	40	7		^36^
IdeS	*Streptococcus pyogenes*		37	6.6		^2^
Procerain B	*Calotropis procera*	28.80	55–60	7–9	9.25	^37^
*Zingibain*	*Z. officinale rhizomes*	33.8	60	7		^38^
Cysteine protease	*Ficus johannis*	25	60	6.50		^39^
Cysteine protease	*Cissus quadrangularis*	39	50	6		^40^
CpCP-1	*Calotropis procera latex*	26.21	30–50	6		^42^
CpCP-2		26.13	30–50	6		
CpCP-3		25.08	30–60	6		
CpCP A	*Calotropis procera latex*	24.82	35	7	4.20	^33^
CpCP C		21.72	35	7.50	9.20	
CpCP D		21.54	35	7.50	9.10	
Cysteine protease	*Ginger rhizome*	36	60	5.50	4.30	^34^

In order to figure out the best method to store rEla for a longer period of time, we incubated the dissolved enzyme at 4, 25 and − 20 °C and also lyophilized enzyme at 4 °C for 100 days. Glycerol at final concentration of 20% was added to half of the – 20 °C samples as it has anti-destruction effect on native protein structures while freezing, by forming hydrogen bonds with water molecules but it caused negative outcome, the enzyme concentration decreased and it showed 15% less activity in comparison with samples kept in − 20 °C without glycerol^52^. Altogether the results indicate that rEla has remarkable stability and enzyme half-life at room temperature is about 100 days.

One of the important features of alkaline proteases is their substrate specificity^32^. Therefore, we investigated substrate specificity of the purified protease with different substrates, and rEla activity was higher when gelatin and collagen were used. Based on our research, no cysteine protease with specificity for collagen substrates has been reported so far. This specific feature along with unique thermostability increases the chance of industrial and medical applications for the enzyme.

It has been noted that alkaline proteases are in need of divalent metal ions such as Ca^2+^ and Mg^2+^ to show their maximum activities. These cation ions role in keeping active conformation of enzyme in high temperatures, is of a great importance, owning to the fact that they protect the protease from thermal denaturation^53^. Supplementary Table S2 represents the effect of several metal ions on rEla and other cysteine proteases in comparison. Among the examined ions, Ca^2+^ increased enzyme activity to 135% at 2 mM concentration, suggesting it as possible rEla cofactor.

Glycerol is an efficient chaperone which is broadly used for in vitro studies. It enhances the stability of proteins through increase in hydration and prevention of protein aggregation^54^. Therefore rEla activity was expected to increase or remain uninfluenced. But as discussed before the remarkable decrease might probably be due to the fact that glycerol has three hydroxyl groups, but doesn’t have a hydrophobic chain and it makes the microenvironment around the enzyme less suitable for catalytic activity^55^. On the other hand as docking result suggests, glycerol binds with allosteric site of the enzyme and might effect on rEla active conformation.

Since non-ionic surfactants such as SDS are very routine additives in detergent industries, the employed enzyme stability in their presence is very important^51^. SDS at 2 and 4% concentration, increased rEla activity up to 386 and 362% respectively. SDS at low concentration attracts sulfhydryl group of hydrogen atom in cysteine residue and increases proteolytic activity. This cysteine probably facilitates the binding of substrate-enzyme reaction^32^. The substantial increase in protease activity at the presence of surfactants is due to their synergetic interaction with the cationic surfactants and therefore, decreasing the positive charge density and reducing the possibility of cationic inhibition of the enzyme active site^56^. On the other hand, due to the existence of hydrophobic environment surrounding the SH groups within the cysteine proteases, nonionic surfactants have better chance to increase interactions with the probable substrates^57^. See the effect of organic solvents and surfactants on catalytic activity of several cysteine proteases on Supplementary Table S3 online.

The enzyme had not any activity in the presence of IAA and IAM, indicating that it belongs to the class of cysteine proteases, whereas IAA and IAM bind covalently with the thiol group of cysteine residue in enzyme catalytic site, the substrate is thereby prevented from binding to the active site. On the other hand, the serine-protease inhibitor PMSF did not have significant inhibitory effect and enzyme retained about 67% of its relative activity after incubating with 2 mM PMSF. As shown in Table 3. β-ME had the opposite effect on rEla in comparison with other cysteine proteases and inhibited the protease slightly at the concentration of 2 mM, however reduced the enzyme activity to 20% at higher concentration (5 mm) suggesting the importance of disulfide bonds in preserving rEla active conformation^58^. In addition, the enzyme was inhibited by EDTA to the extent of 56% and 40% at 2 mm and 5 mm concentration, respectively. Unlike other studied cysteine proteases that EDTA failed to inhibit the enzymes, the observed partial inhibitory effect on rEla cysteine protease might indicate the requirement of Ca^2+^ ions for optimum activity of the enzyme. As EDTA is a chelating agent that binds to calcium ions which are essential for the optimum activity of rEla. rEla cysteine protease seemed to be less resistance to urea than *Ficus johannis* cysteine protease which maintained completely active against 6 mM urea, and lost up to 70% of proteolytic activity at 5 mM concentration of urea.

**Table 3:** Effect of inhibitors on rEla several characterized cysteine proteases.

Proteolytic activity (%)								References
Protein	Concentration	IAA	IAM	PMSF	EDTA	Urea	B-ME	
*Cohnella* sp. A01 cp	2 mM	2	2	66	56	67	86	This study
	5 mM	0	0	12	40	32	20	
IdeS	1 mM	0	0	0	100 <			^2^
Procerain B	40 µM	12						^37^
	50 µM			85				
	1 mM				100			
*CpCP-1*		3.20		96.22	96.33			^33^
CpCP-2		1.76		97.10	99.25			
CpCP-3		2.69		98	96.74			
Ficus johannis cp	1 mM	0		96	105			^39^
	5 mM			93	113			
	6 mM					100		

Competitive inhibitors enhance the Michaelis constant (K_m_), but doesn’t change the maximum velocity (V_max_). Thus, they are assumed to be structural analogues of the substrate competing to take the same active site on the enzyme^59,60^. Therefore, accordingly Leupeptin is a competitive inhibitor of rEla (Fig. 12a). E.64 is an uncompetitive type of inhibitor, which changes both Km and V_max_ (Fig. 12b). Although, uncompetitive inhibitors do not interact with free enzyme, they are capable of merging with the ES complex to decelerate or prevent the product formation^59^. These data support the outcome of docking and MD simulations indicating that Leupeptin binds to active center while E.64 interacts with allosteric site and the enzyme’s conformational changes lead to inactivation.

## Materials and methods

### Chemicals, bacterial strains, plasmids and reagents

PCR Purification Kit and Plasmid purification kit was purchased from Bioneer (Seoul, Korea). DNA purification kit was obtained from PEQLAB*. Cohnella* sp.A1 strain was gathered from the water-waste of shrimp farms located in Choebdeh-Abadan (southwestern of Iran)^61^. *E. coli* strains DH5α and BL21 (DE3), and also Kanamycin resistant pET26b (+) vector were purchased from Invitrogen (Carlsbad, CA, USA). *Pfu* DNA polymerase, IPTG, *Taq* polymerase, proteinase K, trypsin, RNase A, *Nde* I and *Hind* III, DNA ladder, Protein molecular mass marker were provided from Fermentase (Glen Burnie, MD, USA). Kanamycin, L.leucine.p.nitroaniline, PMSF, casein, azo-casein, azo-albumin were acquired from Sigma (Steinheim, USA). All other chemicals were obtained from Merck (Darmstadt, Germany).

### Bioinformatics, homology, template search and sequence alignment

To find homology proteins, BLAST (http://blast.ncbi.nlm.nih.gov/Blast.cgi) was carried out against non-redundant protein database^62,63^. The same process was used for searching homologous sequences of protease in the protein data bank (PDB) to recognize the most similar structures^64^.The alignment was performed with 6 similar sequences from *pfp1* family, to find conserved domains. PRALINE at http://www.ibi.vu.nl/programs/pralinewww/ with the BLOSUM62 substitution matrix and gap penalty of 12 was used for this alignment^65^. The presence of signal peptide and the location of its cleavage site was predicted using SignalP 5.0 server (http://www.cbs.dtu.dk/services/SignalP/). The phylogenetic tree of cysteine protease sequences were analyzed using7 MEGA X program (https://www.megasoftware.net/) and iTOLv^5^ visualizing tool at https://itol.embl.de/^66,67^.

### Secondary and tertiary structure prediction

Software’s developed to predict secondary and 3D protein structures simulate the best and the closest structure to the submitted sequence by applying some algorithms which work based on previously discovered protein structures. To minimize the uncertainty, we compared the results of predictions from at least two commonly used programs for each purpose. Therefore, amino acid sequence of cysteine protease with related PDB file were submitted to the software. Protein secondary structure was predicted using Phyre2 (http://www.sbg.bio.ic.ac.uk/phyre2/)^68^. For 3D structure prediction, SWISS-MODEL (https://swissmodel.expasy.org/), MODELLER 9v7 and I-TASSER server (https://zhanglab.ccmb.med.umich.edu/I-TASSER/) were used^69–71^. The prediction outputs were imported into Chimera 1.14 (http://www.cgl.ucsf.edu/chimera) to evaluate the structures and obtain the optimal energy of best predicted cysteine protease structure^72^. To validate the accuracy of predicted structures, Z-Score point (https://prosa.services.came.sbg.ac.at/prosa.php) was calculated and Ramachandran plot (http://mordred.bioc.cam.ac.uk/~rapper/rampage.php/) was constructed^73^. The active site of the proposed model was predicted using 3DLigandSite (http://www.sbg.bio.ic.ac.uk/3dligandsite/) and evaluated using the Chimera 1.14 program^72,74^.

### Protein–ligand docking studies

The predicted structure was docked with two surfactants (Triton X-100 and glycerol), two inhibitors (leupeptin and E.64) and one substrate (l.leucine.p.nitroaniline) using Molegro Virtual Docker V.6.0 (MVD)^75^. The chemical structures of ligands were obtained from the PubChem. The predicted structure sidechains and obtained ligands were minimized, and the potential cavities (active site and allosteric site) were identified using the built-in cavity algorithm of MVD. For each of the protease-ligand complexes, 20 test runs were performed. The modeled Enzyme was docked with collagen as specific substrate using HADDOCK 2.4^76^. The substrate structure was obtained from RCSB protein data bank (https://www.rcsb.org/). rEla docking results in complexed with collagen and l.leucine.p.nitroaniline were further visualized using Discovery Studio Visualizer v20.1.0.19295 (https://discover.3ds.com/discovery-studio-visualizer-download/).

### Molecular dynamics simulation

MD simulation of the protein–ligand complexes (protease-Triton X-100, protease-E.64 and protease-leupeptin complexes) were performed using GROMACS v 4.6.5 with CHARMM36 all-atom force field^77^. The topologies and parameters of ligands were provided by CGenFF server (https://cgenff.umaryland.edu/) which are compatible with the CHARMM36 all-atoms force field. Protein–ligand complexes were soaked in a cubic box of water molecules. Neutralization of the enzyme charges was carried out by adding Na^+^ and Cl^-^ ions. The system energy was minimized by the steepest descent algorithm to eliminate bad contact and clashes. At last, after releasing all restraints 50 ns MD runs were performed. All bonds were limited by the LINCS algorithm^78^.

### Cloning, expression and purification of the cysteine protease 1759

The gene of cysteine protease 1759 from *Cohnella* sp.A01 was cloned in expression vector pET-26b(+). The gene was amplified using PCR specific forward and reverse primers (forward: 5′-**GGAATTC*CA****TATG*ACCCGGACGAAAAAC-3′ T_m_ = 60 °C and reverse: 5′-**GGG*TTCGA****A*TCAGTGGTGGTGGTGGTGGTGTCGGCGCCCGTACAG-3′ T_m_ = 66 °C) with *Nde* I and *Hind* III recognition sites (shown in italics) respectively. The expression vector and PCR product were then purified using the PCR purification kit and digested with restriction enzymes mentioned above. Ligation of fragments and vector was carried out by T_4_ DNA ligase and the recombinant vector was transformed into a proliferative competent host (*E. coli* DH5α). Recombinant pET26b(+) was then transferred into *E. coli* BL21 (DE3).The bacteria’s grown cell (*E. coli* BL21) containing recombinant vector was cultured and incubated in 5 ml Luria–Bertani (LB) broth for 16 h at 37 °C. One ml of this culture was inoculated in 50 ml of LB broth containing 50 μl kanamycin (1 mM) and incubated at 37 °C. The culture was then shaken at 180 rpm to reach the optimal density (OD) of 0.4–0.6. To induce expression, IPTG (with final concentration of 1 mM) was added to the media and it was incubated for 16 h (overnight) at 27 °C with 150 rpm. Since the intended protease is an intracellular enzyme, the cells were harvested by centrifugation at 8000×*g* for 20 min at 4 °C and the sediment was resuspended in 4 ml lysis buffer containing 300 mM NaCl, 50 mM NaH_2_PO_4_, 68 mg Imidazole and 0.05% Tween 20 with final pH 8. Disintegration of cells was carried out by sonication on ice with 7 × 45 s pulses followed by 60 s rest between cycles. The mixture was again centrifuged at 8000×*g* for 40 min at 4 °C and the supernatant was collected for purification of the enzyme. The enzyme was purified using Ni–NTA Sepharose affinity chromatography, at 4 °C. A dialyzed sample (in potassium phosphate 50 mM, pH 8) was then used to track the cysteine protease on 12.5% sodium dodecyl sulfate–polyacrylamide gel electrophoresis.

### Zymography

Casein zymography was carried out on SDS-PAGE. The resolving gel was copolymerized with casein 1%. After 16 h running, the gel was carefully removed and incubated in potassium phosphate buffer (pH 8, 50 mM) and Ca^2+^ (2 mM) overnight. The gel was then stained with Coomassie Blue R-250 for 15 min. Clear bonds on a blue background indicates the cysteine protease activity.

### rEla resistance against proteases

The stability of the rEla in presence of proteases including trypsin and proteinase K was investigated by incubation of the enzyme and each of proteases (with protease to substrate ratio of 1:20 v/v) for 15 min at 37 °C. Reactions were then analyzed by SDS-PAGE.

### Protease assay

The protease activity was determined using casein 1%. 50 µl of dialyzed enzyme was added to 70 µl of casein 1% in the same buffer. The enzyme co-factor, CaCl_2_ with final concentration of 2 mM was added to the solution. The mixture was then incubated at 50℃ for 30minutes. To terminate the reaction, 125 µl of trichloroacetic acid 15% was used. To precipitate the non-hydrolyzed casein, the tube content was centrifuged at 10,000×*g* for 5 min at 4 °C. Blank contained reaction cocktail without enzyme. The supernatant absorbance was measured at 280 nm and the amount of hydrolyzed proteins was assessed using tyrosine as a standard. One unit of enzymes activity was defined as the amount of enzyme that can produce 1 µmol of tyrosine per minutes at pH 8 and 50 °C.

### Evaluation of temperature and pH on the cysteine protease activity and stability

To determine the cysteine protease temperature profile, enzyme’s activity was measured in temperature ranges from 10 to 90 °C. For estimating thermostability in different temperatures, enzyme was incubated in 10, 20, 30, 40, 50, 60, 70 and 80 °C for 90 min and the residual activity was observed. In another experiment to analyze thermal stability of cysteine protease, it was incubated in 50, 70, 90℃ for 3 h and the enzyme activity was measured every hour at optimum temperature. In order to assess the effect of pH on cysteine protease activity, reaction buffer (50 mM) was prepared in various pH 2–14 (composed of acetate, phosphate and glycine) to find the optimum enzyme activity. For pH stability, enzyme was incubated in 5 mM mixed buffer with different pH (3–12) for 90 min and afterward the protease activity was calculated in pH 8 and in addition pH stability was evaluated by incubating the enzyme in 5 mM mixed buffer at pH values of 5 and 11 for 3 h and the measuring its activity in optimal conditions every hour.

### Kinetic and thermodynamic parameters study

Casein concentration of 1.25 to 80 mg/ml were used to determine the kinetic parameters of cysteine protease and the Michaelis–Menten plot was drawn in GraphPad Prism 6 to calculate Maximum velocity (Vmax) and Michaelis–Menten constant (Km) values.

The thermodynamic parameters of rEla including ΔH^*^, ΔG^*^ and ΔS^*^ values were calculated as follow:

1 $${{k}_{cat}=\frac{{K}_{B}T}{h}.K}_{cat}^{*}$$

2 $$\Delta {G}^{*}=-RTLn\left({K}_{cat}^{*}\right)$$

3 $$\Delta {H}^{*}={E}_{a}-RT$$

4 $$\Delta {G}^{*}= \Delta {H}^{*}-T\Delta {S}^{*}$$

where, K_B_ is the Boltzmann constant (i.e. R/N, which is 1.38 × 10^−23^ J/K), T is the temperature in Kelvin, ℏ is the Planck constant (6.63 × 10^−34^ J/mol K), R is the gas constant (8.314 J/K mol).

To determine the irreversible thermo-inactivation parameters, according to the temperature stability plot the following equation was used, where A_0_ is the initial enzyme activity, A_t_ is the enzyme activity at intended time, k is the inactivation rate constant:

5 $$Ln\frac{{A}_{t}}{{A}_{0}}=-kt$$

To assess thermodynamic properties (ΔE^#^, ΔH^#^, ΔG^#^, ΔS^#^), the Arrhenius plot was designed for irreversible thermal denaturation of enzyme at 50, 70, and 90℃. The thermodynamic parameters were calculated by Eqs. (6–9) where k_B_ is Boltzmann constant, h is Planck constant and k_in_ is the inactivation rate constant:

6 $$\mathrm{Ln}\left({k}_{in}\right)=-{E}_{a }/RT$$

7 $$\Delta {G}^{\#}=-RT ln(\frac{{k}^{in}h}{{k}_{B}T})$$

8 $$\Delta {H}^{\#}=\Delta {E}_{a}-RT$$

The Gibbs free energy of inactivation (ΔG^#^) of the protease was calculated from:

9 $${\Delta }G^{\# } = {\Delta }H^{\# } - T{\Delta }S^{\# }$$

Moreover, enzyme half-life at 50, 70 and 90 °C was estimated using the Eq. (10).

10 $${t}_{1/2}=ln(2)/{k}_{in}$$

### Long-term storage of rEla

In order to study the enzyme long-term stability at 25 °C, 4 °C and − 20 °C enzyme was incubated at mentioned temperatures for 100 days and its activity was measured after 30, 60 and 100 days and it was compared to lyophilized sample to figure out the best method of storing the enzyme. To keep enzyme at − 20 °C, we considered two samples one with glycerol added to the buffer (final concentration 20%) to avoid protein damage and denaturation and one without it^52^.

### Protease specificity

In order to determine protease specificity, enzyme activity was measured using azocasein, albumin, gelatin, collagen and l.leucine.p.nitroaniline as substrates. In the case of azocasein, 90 µl the substrate 1% and 10 µl of enzyme solution was incubated for 30 min in optimum temperature and pH. The reaction was then terminated by adding cold TCA 15%. The solution was centrifuged 15 min in 4000×*g*. Absorbance of the supernatant was determined in 440 nm. In assay with l.leucine.p.nitroaniline, 30 µl substrate (10 mM), 100 µl protease, 120 µl distilled water and 250 µl Tris–HCL (100 mM) was incubated for 30 min in optimum temperature and pH. To cease the reaction, 100 µl of acetic acid 30% (w/v) was used. Samples were centrifuged 10 min in 4000×*g*. Absorbance of the supernatant was determined in 410 nm. Assays with albumin, collagen and gelatin were also carried out the same way as explained for azocasein.

### Effect of metal ions, surfactants, organic solvents and chemical compounds on enzyme activity

The reaction mixture was prepared in the present of ions such as NaCl, KCl, MgCl_2_, LiCl, CaCl_2_, BaCl_2_, AlCl_3_, MnCl_2_ CoCl_2_, CuSO_4_, ZnSO_4_ (2 and 5 mM), surfactants like Tween 20, Tween 80, Triton X-100, SDS (2 and 5 mM), organic solvents including methanol, ethanol, isopropanol, acetone, glycerol (10 and 20% v/v) and chemical compounds IAM, PMSF, IAA, β-ME and EDTA (2 and 5 mM). The enzyme and casein 1% solution was considered as blank. Enzyme residual activity was determined against blank.

### Specific inhibitors

Enzyme’s activity was analyzed in the presence of cysteine proteases specific inhibitors such as Leupeptin and *trans*-Epoxysuccinyl-l-leucylamido (4-guanidino) butane (E.64) (2.5 and 5 µM), in different concentration of casein (1.25–70 mg/ml). The type of inhibition was assessed using Lineweaver–Burk plot.

## Supplementary Information

Supplementary Information.
