# Supplementary material for: Cohnella 1759 cysteine protease shows significant long term half-life and impressive increased activity in presence of some chemical reagents
Source: Sci Rep. 2021 Feb 25;11:4573. doi: 10.1038/s41598-021-84267-w (PMC7907070; doi:10.1038/s41598-021-84267-w)
Supplement: Supplementary file 1 — Supplementary Information. [file 41598_2021_84267_MOESM1_ESM.docx]

***Cohnella* 1759 Cysteine Protease shows significant long term half-life and impressive increased activity in presence of some chemical reagents**

Rayan Saghian^#^, Elham Mokhtari^#^, Saeed Aminzadeh^*^

Bioprocess engineering group, Institute of Industrial and Environmental Biotechnology, National Institute of Genetic Engineering and Biotechnology (NIGEB), Tehran, Iran

*Corresponding Author; E-mail: [aminzade@nigeb.ac.ir](mailto:aminzade@nigeb.ac.ir)

^#^Co-first authors

| **Source** | **Substrate** | **K_m (mM)_** | **k_cat_ (s^-1^)** | **k_cat_ / K_m_** | **Ref.** |
| --- | --- | --- | --- | --- | --- |
| rEla | Casein | 21.93 | 8.6 | 4.09×10^2^  M^-1^S^-1^ | This study |
| Protrase 1147 | Casein | 13.72 | 3.143×10^-3^ | 0.381M^-1^S^-1^ | ^1^ |
| Babesia bigemina | Synthesized | 59.0 µg/ml**^-1^** | 0.306 | 5.19×10**^3^** | ^2^ |
| Fluke Fasciola hepatica | Casein | 160µg/ml**^-1^** | 1.0 | 0.06×10**^2^** | ^3^ |
| Ficin | Casein | 13.7 | 32.9 | - | ^4^ |
| ZCPG | Casein | 0.5±0.03 µg | - | - | ^5^ |
| Taenia solium CP | Z-Phe-Arg-AFC | 7.0 × 10^-3^ | 1.98×10^5^ | 2.84×10^9^ | ^6^ |

**Supplementary Table S1.** Kinetic parameters of some characterized cysteine proteases.

**Supplementary Table S2**. The effect of different final concentration of some metal ions on some cysteine proteases.

| **protein** | **Salt**  **concentration** | **Ca^2+^** | **Co^2+^** | **Mg^2+^** | **Ba^2+^** | **Al^3+^** | **Cu^2+^** | **Zn^2+^** | **Na^+^** | **K^+^** | **Li^+^** | **Mn^2+^** | **References** |
| --- | --- | --- | --- | --- | --- | --- | --- | --- | --- | --- | --- | --- | --- |
| *Cohnella* sp.A01 cp | 2mM  5mM | 135%  44% | 22%  9% | 65%  0 | 27%  40% | 4%  50% | 30%  27% | 0 | 86%  89% | 71%  36% | 55%  0 | 16%  0 | This study |
| *Zingiber montanum* cp | 1mM |  | 30% |  |  | 100% | 40% | 30% | 100% |  |  | 100% | ^5^ |
| Ficin (EC 3.4.22.3) | 1mM  5mM | 83%  69% |  | 80%  61% | 97%  91% |  | 3%  0 | 49%  26% |  |  |  |  | ^4^ |
| Cissus quadrangularis cp | 10mM | 72% |  | 86% |  |  |  |  |  |  |  |  | ^7^ |
| *Ficus johannis*  cp | 0.75mM  1mM  10mM | 51.8 |  | 91 | 100 |  | 51 |  | 80 | 100 | 100 |  | ^8^ |

**Supplementary Table S3.** The effect of organic solvents and surfactants on catalytic activity of several cysteine proteases.

|  | **concentration** | **Organic solvents** | | | | | **concentration** | **Surfactants** | | | | References | |
| --- | --- | --- | --- | --- | --- | --- | --- | --- | --- | --- | --- | --- | --- |
|  |  | Methanol | Ethanol | Iso-propanol | Glycerol | Acetone |  | Tween20 | Tween80 | Triton X-100 | SDS |  |  |
| *Cohnella* sp.A01 cp | 10%  20% | 72  54 | 90  57 | 0  0 | 34  8 | 0  0 | 1%  4%  8% | 419  162  5 | 117  268  5 | 472  488  80 | 386  362  57 | This study |  |
| Cissus quadrangularis cp |  | 45 | 36 | 63 |  | 82 |  |  |  |  |  | ^7^ |  |
| *Ficus johannis*  cp | 50% | 101 | 100 | 70 |  |  | 0.5%  6% |  |  | 75 | 70 | ^8^ |  |

Supplementary fig. S1. Graphical abstract. Created in [BioRender.com](https://biorender.com/).


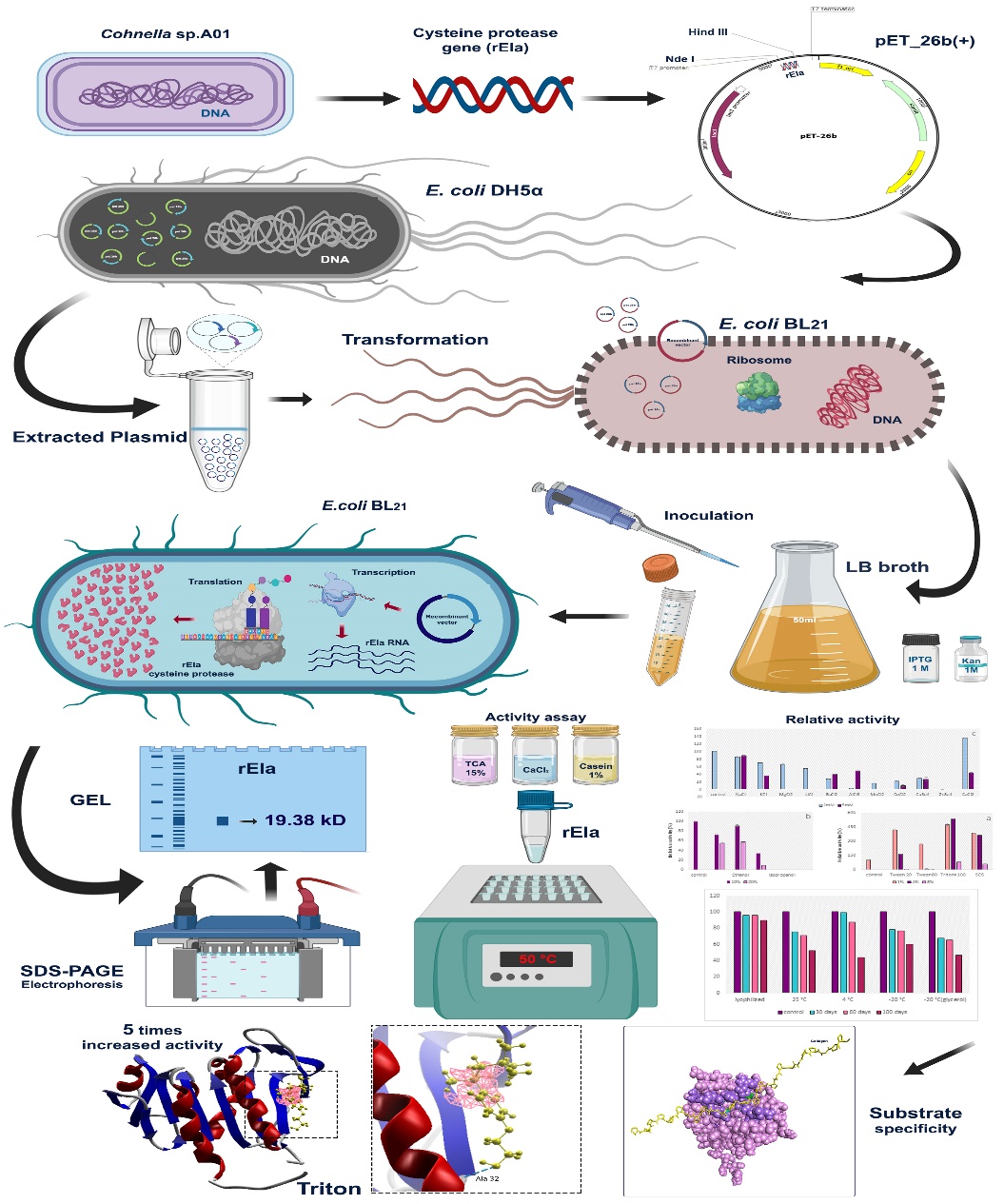


Supplementary fig. S2. Prediction of the signal peptide of the enzyme. SignalP software suggested rEla doesn’t have any signal peptide.


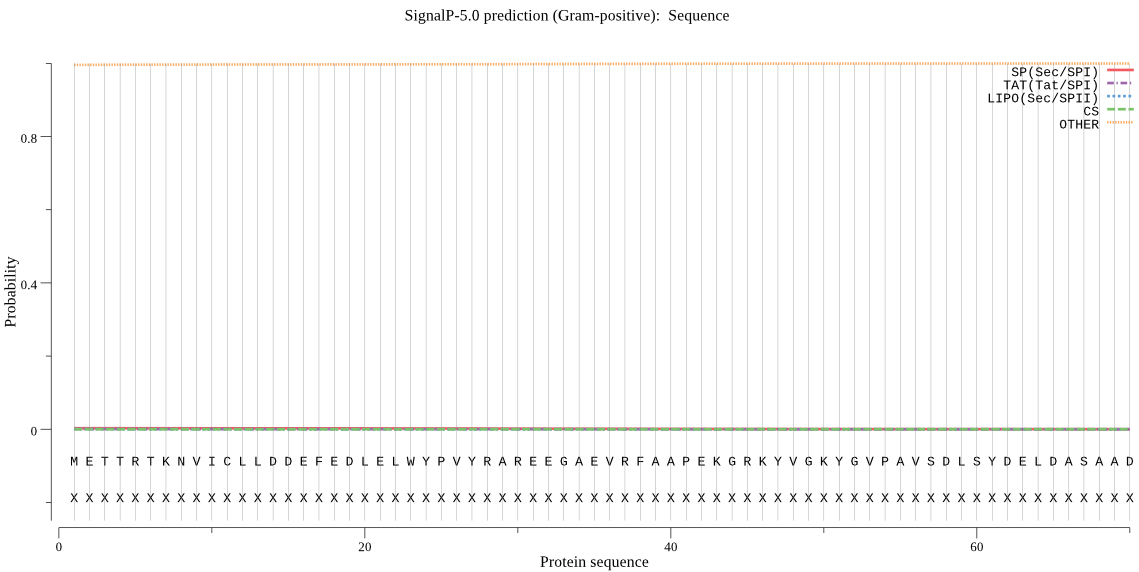


Supplementary fig. S3. ProSA energy plot of the residues. As shown most of the residues have negative energy.


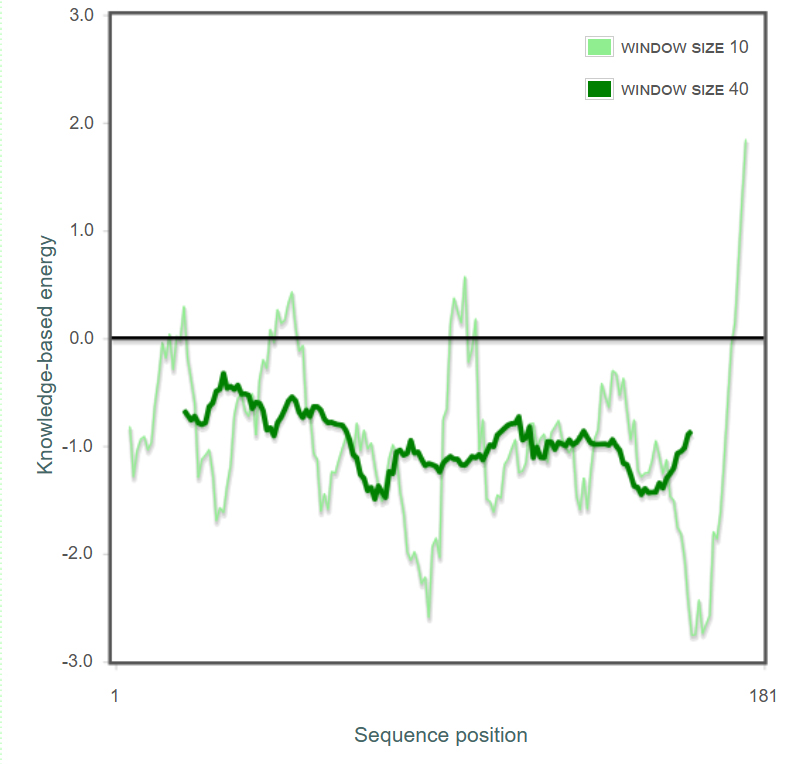


Supplementary fig. S4. Full-length (zymography, expression, long-term stability and resistance against proteases) gels of Fig.8


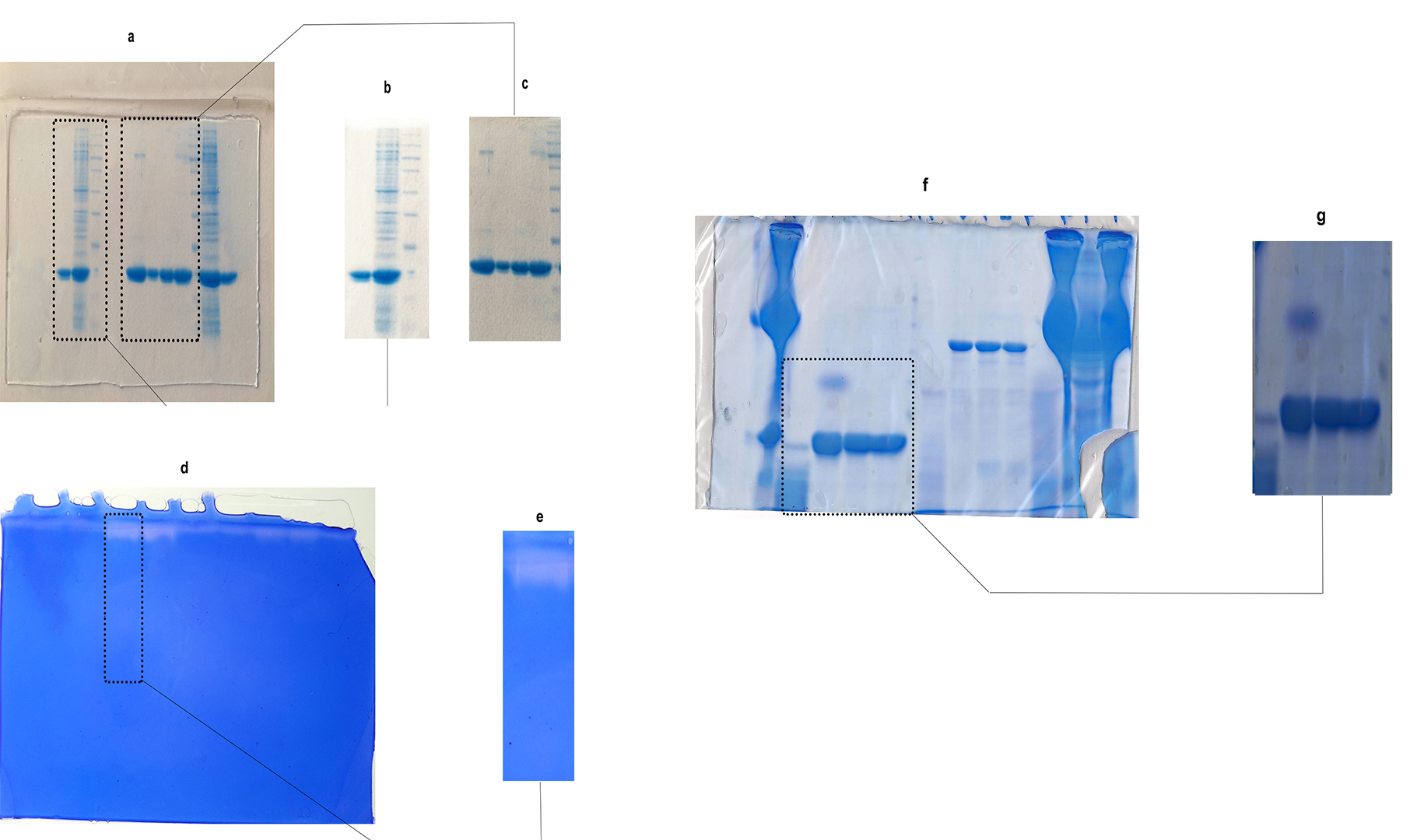


Supplementary fig. S5. (a) Michaelis-Menten plot of rEla. The plot was constructed using “GraphPad prism 6”. (b) Thermo-inactivation of the rEla Ln at 50, 70 and 90˚C.


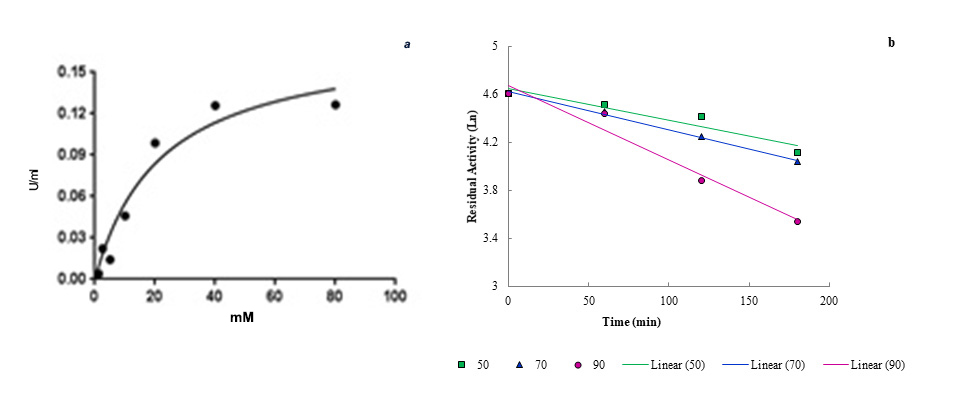


Supplementary fig. S6**.** The enzyme activity in presence of different concentrations of specific inhibitors: Leupeptin and E.64.


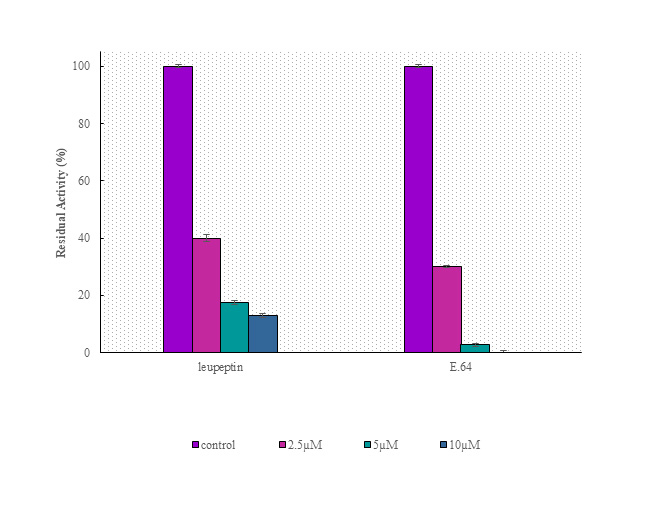


References

1. Tarrahimofrad, H. *et al.* Structural and biochemical characterization of a novel thermopilic Coh01147 protease. *PLoS One* **15**, 1–29 (2020).

2. Martins, T. M., do Rosário, V. E. & Domingos, A. Expression and characterization of the Babesia bigemina cysteine protease BbiCPL1. *Acta Trop.* **121**, 1–5 (2012).

3. Hemici, A. A., Benerbaiha, R. S. & Bendjeddou, D. Purification and biochemical characterization of a 22-kDa stable cysteine- like protease from the excretory-secretory product of the liver fluke Fasciola hepatica by using conventional techniques. *J. Chromatogr. B* (2017) doi:10.1016/j.jchromb.2017.10.049.

4. Homaei, A., Stevanato, R. & Hemmati, R. Author ’ s Accepted Manuscript. *Biocatal. Agric. Biotechnol.* (2017) doi:10.1016/j.bcab.2017.04.008.

5. Jamir, K. & Seshagirirao, K. Purification, biochemical characterization and antioxidant property of ZCPG, a cysteine protease from Zingiber montanum rhizome. *Int. J. Biol. Macromol.* (2017) doi:10.1016/j.ijbiomac.2017.08.078.

6. Baig, S., Damian, R. T., Molinari, J. L. & Tato, P. Purification and characterization of a metacestode cysteine proteinase from Taenia solium involved in the breakdown of human IgG. 411–416 (2005) doi:10.1017/S0031182005007821.

7. Sakthivel, M. *et al.* Antibacterial cysteine protease from Cissus quadrangularis L . *Int. J. Biol. Macromol.* (2017) doi:10.1016/j.ijbiomac.2017.05.107.

8. Afsharnezhad, M., Shahangian, S. S. & Sariri, R. PT. *Int. J. Biol. Macromol.* #pagerange# (2018) doi:10.1016/j.ijbiomac.2018.10.006.
